# Cell-Free Phospholipid Biosynthesis by Gene-Encoded Enzymes Reconstituted in Liposomes

**DOI:** 10.1371/journal.pone.0163058

**Published:** 2016-10-06

**Authors:** Andrew Scott, Marek J. Noga, Paul de Graaf, Ilja Westerlaken, Esengul Yildirim, Christophe Danelon

**Affiliations:** Department of Bionanoscience, Kavli Institute of Nanoscience, Delft University of Technology, Van der Maasweg 9, 2629 HZ, Delft, The Netherlands; University of Cambridge, UNITED KINGDOM

## Abstract

The goal of bottom-up synthetic biology culminates in the assembly of an entire cell from separate biological building blocks. One major challenge resides in the *in vitro* production and implementation of complex genetic and metabolic pathways that can support essential cellular functions. Here, we show that phospholipid biosynthesis, a multiple-step process involved in cell membrane homeostasis, can be reconstituted starting from the genes encoding for all necessary proteins. A total of eight *E*. *coli* enzymes for acyl transfer and headgroup modifications were produced in a cell-free gene expression system and were co-translationally reconstituted in liposomes. Acyl-coenzyme A and glycerol-3-phosphate were used as canonical precursors to generate a variety of important bacterial lipids. Moreover, this study demonstrates that two-step acyl transfer can occur from enzymes synthesized inside vesicles. Besides clear implications for growth and potentially division of a synthetic cell, we postulate that gene-based lipid biosynthesis can become instrumental for *ex vivo* and protein purification-free production of natural and non-natural lipids.

## Introduction

Life as we know it is compartmentalized: a continuous membrane encloses the cytoplasm protecting it from the environment and specifying a unit of evolutionary selection. This cellular envelop is primarily made of phospholipids that, together with specific proteins, control shape transformation and regulate the ionic and molecular exchanges with the external medium. Several laboratories are now attempting to construct a minimal, albeit sufficient, cell starting from purified components derived from existing organisms [1–6]. Given the central roles played by the cellular membrane, an important milestone in the roadmap for creating a synthetic cell that can grow and divide is the *de novo* synthesis of membrane constituents from internally produced enzymes.

An attractive metabolic pathway for lipid biosynthesis is through diacyl-phosphatidic acid (PA), the universal precursor of glycerophospholipids in bacteria [7,8]. The pathway for PA synthesis in *E*. *coli* entails two acyltransferase enzymes: the glycerol-3-phosphate (G3P) acyltransferase (GPAT) and the lysophosphatidic acid (LPA) acyltransferase (LPAAT) [8,9]. The enzyme GPAT is an integral membrane protein that uses G3P and either acyl-CoA (CoA, coenzyme A) or acyl-ACP (ACP, acyl carrier protein) substrates to generate 1-acyl-*sn*-glycerol 3-phosphate products (LPA). In a subsequent enzymatic reaction the LPA and another acyl-CoA/acyl-ACP are converted into 1,2-diacyl-*sn*-glycerol 3-phosphate (PA) by the membrane-bound enzyme LPAAT. In the cellular context of *E*. *coli*, the GPAT and LPAAT enzymes are then complemented by a few others to modify the lipid headgroup and produce phosphatidylglycerol (PG), a bilayer-forming anionic lipid, and phosphatidylethanolamine (PE), a zwitterionic lipid, together representing the largest fraction of the *E*. *coli* inner membrane lipidome [10,11].

To date several attempts have been made to stimulate compartment growth in phospholipid vesicles by using purified acyltransferase enzymes [12–14]. More recently, the activity of the GPAT and LPAAT enzymes synthesized from a reconstituted *in vitro* transcription-translation (IVTT) system has been demonstrated in separate reactions [15]. However, these two enzymes failed to work in the same environment and conflicting oxidative-reductive requirements for proper enzymatic activities were invoked [15].

Hereby, we report on the cell-free production and functional liposome reconstitution of multiple lipid-synthesizing enzymes in the PURE system [16], a well-defined IVTT system, starting from the genes and the phospholipid precursors G3P and acyl-CoA. Protein synthesis and lipid biogenesis, two essential functions for cell homeostasis, occurred in a single environment demonstrating the compatibility of these biological modules for further integration into a semi-synthetic minimal cell. We validated the use of liquid chromatography mass spectrometry (LC-MS) as a powerful analytical technique to quantify the amount of synthesized lipids. Focusing on the two-step acyl transfer reaction, we first demonstrated that the co-expressed GPAT and LPAAT enzymes enabled the synthesis of the membrane constituent 1,2-diacyl-*sn*-glycerol 3-phosphate in a single-pot reaction, including when compartmentalized inside liposomes. Capitalizing on the *de novo* synthesis of PA we then reconstituted the entire *E*. *coli* metabolic pathways to convert the PA headgroup into PE and PG lipids. Our work provides a new experimental framework to build up a genetically controlled synthetic cell where the compartment is produced *in situ* from simple biochemical precursors.

## Results

### *In vitro* synthesis and liposome reconstitution of the GPAT and LPAAT enzymes

**Fig 1:**
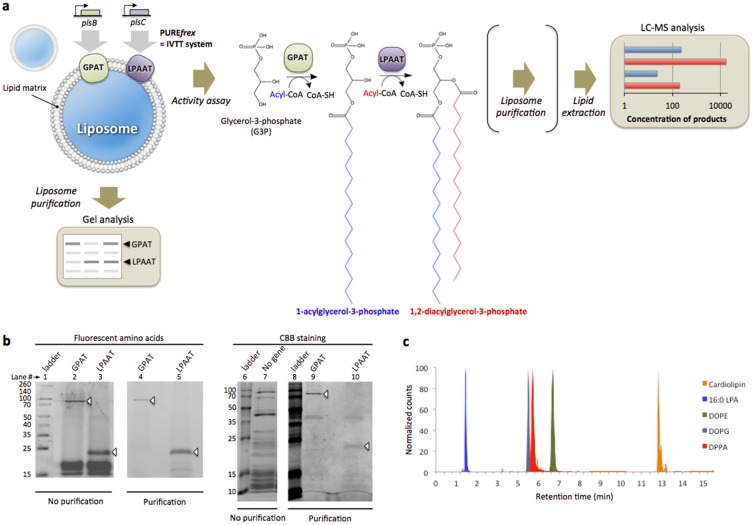
Overview of methods for cell-free transcription-translation of acyltransferase enzymes. (**a**) The genes *plsB* and *plsC* coding for the GPAT and LPAAT enzymes, respectively, were expressed by *in vitro* transcription translation (IVTT) in the presence of SUVs. Spontaneously assembled proteoliposomes containing synthesized GPAT and LPAAT proteins were isolated by ultracentrifugation (floatation method) and the protein content was analysed by SDS-PAGE. Activity assays were performed by adding the phospholipid precursors G3P and acyl-CoA (shown in the reaction scheme is palmitoyl-CoA, p-CoA) either before or subsequent to IVTT reaction. Biosynthesis of 1,2-diacylglycerol-3-phosphate (here DPPA) occurs in a two-step acyl transfer reaction catalysed by the GPAT and LPAAT enzymes. The intermediate product 1-acylglycerol-3-phosphate (here 16:0 LPA) and two free CoA molecules are also formed. After reaction the lipid fraction was extracted and assayed by LC-MS. To quantify the enrichment of vesicles with synthesized lipids, liposomes were purified by immobilization on beads before the lipid extraction step. (**b**) Cell-free expression of either the *plsB* or *plsC* gene (no gene as negative control) occurred for 3 h at 37°C in the presence of 100-nm SUVs and of GreenLys reagent (tRNA-loaded fluorescent amino acid) for fluorescence labelling of translation products. Reconstituted proteoliposomes were purified and membrane-integrated proteins separated by SDS-PAGE were visualized with coomassie brilliant blue (CBB) staining and fluorescence scanning. As shown with CBB staining the PURE system background proteins (lane 7) can efficiently be eliminated by purification, while the GPAT and LPAAT protein bands were co-purified with the SUVs (lanes 9,10). Isolation of acyltransferase enzymes is also visible on the fluorescence scan (lanes 4,5). The lower bands on lanes 2,3 correspond to background signal from the GreenLys reagent. (**c**) Normalized chromatogram of lipids as measured by LC-MS operating in multiple reaction monitoring (MRM) mode with negative polarity. In this example, 16:0 LPA, DOPG, DPPA, DOPE and cardiolipin were clearly resolved. Since the quaternary amine has a permanent positive charge, DOPC is not well detected in the negative mode. It was also possible to detect 18:1 LPA, DOPA, DPPS, DPPG and DPPE (Figure A in S1 File).

We first verified that cell-free expression in the PURE system of the *plsB* and *plsC* genes, respectively encoding for the GPAT and LPAAT proteins, led to full-length proteins. The *E*. *coli* GPAT and LPAAT enzymes were separately synthesized from their respective DNA template and the translation products were analysed by SDS-PAGE and fluorescence gel imaging. A fraction of tRNA pre-loaded with a fluorescently labelled lysine was supplemented in the IVTT reaction to facilitate detection of the synthesized protein over the PURE system background. The *in vitro* produced GPAT and LPAAT proteins were visualized as distinct bands at around 83 kDa and 27 kDa (Fig 1b) as previously reported [17,18].

It is known that GPAT is an integral membrane protein [19] and LPAAT is thought to be a membrane-anchored protein [18]. We thus examined the ability of the synthesized enzymes to associate to the liposome membrane. Preformed small unilamellar vesicles (SUVs) composed of DOPC/DOPE/DOPG/cardiolipin were supplemented in the IVTT reaction carrying out the expression of the *plsB* or/and *plsC* genes, whose encoded proteins inserted into the SUV membranes in an inside-out configuration (i.e. assuming unidirectional incorporation of the proteins after which their cytosolic side faces the outside of the liposomes) (Fig 1a). The proteoliposomes were purified from the bulk fraction by ultracentrifugation and the protein content associated to the liposome membrane was analysed by SDS-PAGE (Fig 1b). The PURE system proteins could efficiently be eliminated, whereas both GPAT and LPAAT enzymes co-purified with the liposomes (Fig 1b), suggesting that these two proteins exhibit the structural properties for stable co-translational insertion or anchoring to the membrane. The process of membrane incorporation is passive, in that it does not require a translocation machinery.

### Both synthesized GPAT and LPAAT enzymes are active when co-reconstituted in liposomes

Having established that the full-length GPAT and LPAAT proteins can be synthesized in the PURE system and be incorporated in the membrane of liposomes we then explored the potential of mass spectrometry (MS) combined with liquid chromatography (LC) to detect the products of the GPAT and LPAAT enzymatic reactions in a background of the lipids comprising the liposomes and to quantify their amounts. A typical chromatogram, where one can clearly distinguish the enzymatic products palmitoyl lysophosphatidic acid (LPA(16:0/0:0), referred as 16:0 LPA) and 1,2-dipalmitoyl-*sn*-glycero-3-phosphate (PA(16:0/16:0), referred as DPPA) in a cardiolipin/DOPX (where PX = PC, PE, PG headgroups) lipid matrix, is shown in Fig 1c. The detection sensitivity of the combined LC-MS was estimated to 0.25 pmol. for 16:0 LPA and DPPA (Figure B in S1 File), which is better than usually reported via radioactive elements separated by thin layer chromatography. The method was expanded to detect the synthesized lipids oleoyl lysophosphatidic acid (LPA(18:1/0:0), referred as 18:1 LPA), 1,2-dioleoyl-*sn*-glycero-3-phosphate (PA(18:1(9Z)/18:1(9Z)), referred as DOPA), 1,2-dipalmitoyl-*sn*-glycero-3-phosphatidylethanolamine (PE(16:0/16:0), referred as DPPE) and 1,2-dipalmitoyl-*sn*-glycero-3-phospho-(1'-*rac*-glycerol) (PG(16:0/16:0), referred as DPPG).

**Fig 2:**
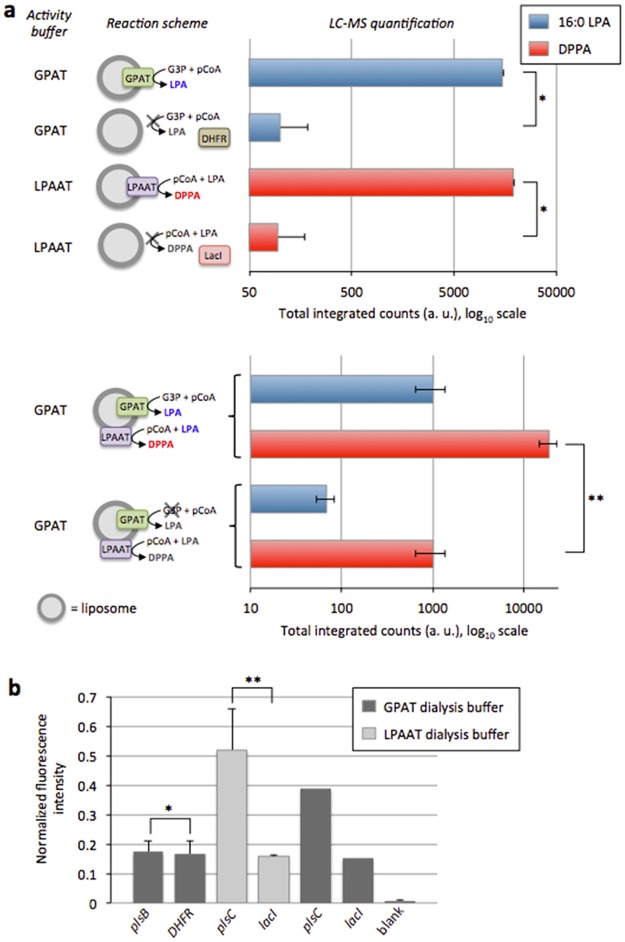
Two-step acyl transfer reaction mediated by cell-free synthesized GPAT and LPAAT enzymes. (**a**) LC-MS analysis of the GPAT and LPAT reaction products. The lipid precursors G3P and palmitoyl-CoA (p-CoA), or p-CoA and 16:0 LPA (66.6 μM each, except in two-enzyme cascade experiments, where p-CoA concentration was 133.3 μM) were added after the IVTT reactions performed in the presence of SUVs. The two enzymes were assayed separately in their respective activity buffer or together in the reducing buffer known to support GPAT activity. Negative controls in GPAT and LPAAT activity buffers were performed using the *DHFR* and *LacI* genes. For combined GPAT and LPAAT reactions, controls were conducted without G3P. Error bars in single-enzyme experiments are s.e.m. from multiple measurements of one sample. In the GPAT and LPAAT co-expression experiments data are mean and s.e.m. across four independent samples; For each repeat the sample was injected multiple times, the average value of the different injections was calculated and data are reported as the mean and standard error of independent trials. Student *t*-test analysis: *P<0.015, **P<0.025. (**b**) Acyltransferase activity as measured using a fluorescence-based assay in which released CoA reacts with a fluorogenic substrate. Negative controls for GPAT and LPAAT activity were performed using the *DFHR* and *LacI* genes, respectively. DTT was dialysed out after the IVTT reaction to create the non-reducing conditions compatible with the assay. Blank was measured from the buffer included in the fluorescence-based CoA assay kit. Data are mean values and s.e.m. of two independent experiments. Student *t*-test analysis: *Difference statistically not significant, **P<0.23.

Next, we sought to assay the activity of the two enzymes. Gene expression and lipid synthesis were first examined sequentially. The enzymes GPAT and LPAAT were individually assayed in specific buffer conditions known to support their activity [15]. GPAT catalysed the formation of 16:0 LPA starting from G3P and palmitoyl-CoA substrates, while LPAAT converted palmitoyl-CoA and 16:0 LPA into DPPA. The formation of enzymatic products was quantitatively detected by LC-MS (Fig 2a). In addition, we used a fluorescence-based acyltransferase activity assay to monitor the accumulation of released CoA molecules through enzymatic reaction of the GPAT and LPAAT proteins (Fig 2b). As anticipated GPAT activity could not be observed since the reducing agent DTT had to be removed before triggering the reactions (see Methods). However, a clear increase of fluorescence signal was detected when LPAAT proteoliposomes were incubated with palmitoyl-CoA and LPA substrates in LPAAT-specific buffer and, interestingly, in the GPAT-specific buffer too (Fig 2b).

The two-enzyme cascade reaction was analysed using inside-out proteoliposomes containing both synthesized GPAT and LPAAT proteins. The proteoliposomes were supplied with G3P and palmitoyl-CoA and incubated in the GPAT activity buffer. In contrast to what has previously been reported [15], we found that the output lipid DPPA was successfully produced, demonstrating that LPAAT can also be active in a reducing environment (Fig 2a). Because the LPA produced by the GPAT enzyme is subsequently used as a substrate by LPAAT in the cascade reaction, it does not accumulate and its detected concentration is less than that in a GPAT-only reaction (Fig 2a).

### Combined gene expression and enzyme-catalysed lipid biosynthesis in a one-pot reaction

**Fig 3:**
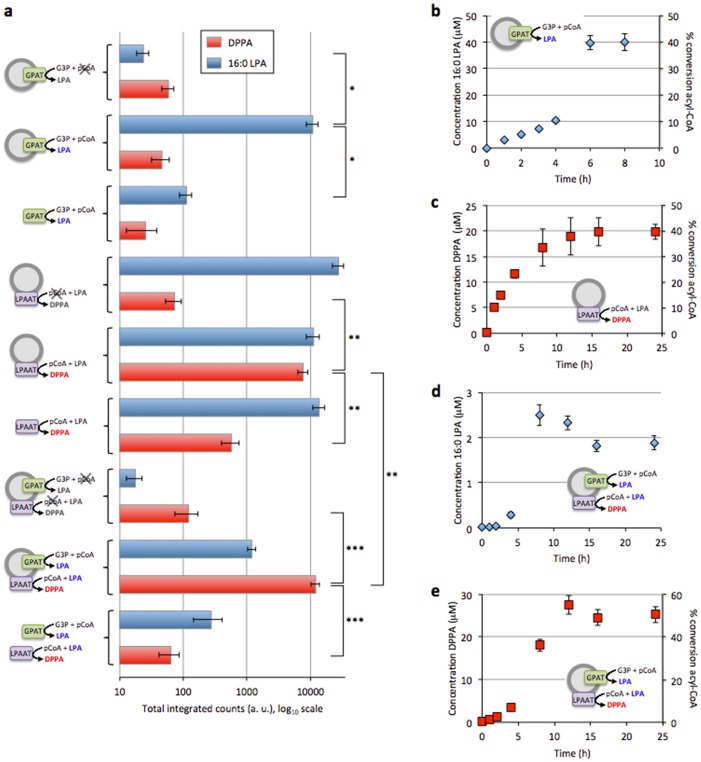
One-pot IVTT and acyl transfer reactions. The GPAT and LPAAT enzymes were either produced separately or concurrently in the presence of G3P and p-CoA substrates. The generated lipid products 16:0 LPA and DPPA were detected by LC-MS. (**a**) End-point measurements of 16:0 LPA and DPPA synthesized under various experimental conditions. Substrate concentrations were 500 μM G3P, 100 μM p-CoA and 100 μM 16:0 LPA. Individual and combined enzymatic reactions were carried out with (inside-out configuration) or without 400-nm liposomes during overnight incubation at 37°C. Samples with liposomes and without p-CoA served as a negative control. Both acyltransferase enzymes showed reduced activity in the absence of SUVs. Higher yield of DPPA is obtained by two-step acyl transfer catalysed by the GPAT and LPAAT enzymes co-reconstituted in proteoliposomes. Data represent mean and s.e.m. of three independent experiments. For each repeat the mean of multiple sample injections was calculated and data are reported as the mean and standard error of three independent trials. Student *t*-test analysis: *P<0.1, **P<0.12, ***P<0.012. (**b-e**) Kinetic of acyltransferase activity in single-enzyme and two-enzyme modes. For each reaction scenario the percentage of acyl-CoA conversion to final product is also indicated. Substrate concentrations were 500 μM G3P and 100 μM p-CoA (**b**), 500 μM G3P, 50 μM p-CoA and 50 μM 16:0 LPA (**c**), 500 μM G3P and 100 μM p-CoA (**d,e**). Produced 16:0 LPA does not accumulate beyond 3 μM in the two-step acyl transfer scheme (**d**) since it is consumed in the second enzymatic reaction. When GPAT and LPAAT are co-expressed, production of DPPA is initially limited by GPAT activity but then it reaches higher concentration (**e**) than with LPAAT only starting from purified LPA and p-CoA precursors (**c**). Each data point is mean and s.e.m. of two independent sample preparations. For each replicate the mean of two sample injections was calculated and data are reported as the mean and standard error of independent preparations.

In light of this new result, we tested whether the GPAT and LPAAT enzymes could be synthesized from their DNA, insert into the membrane of preformed vesicles and generate lipid products, all in a single-pot reaction. Both 16:0 LPA and DPPA products were measured, showing that gene expression and lipid biosynthesis can successfully be integrated in the PURE system (Fig 3). In a cascade reaction, about 10% of LPA was measured relative to the amount detected with GPAT-only proteoliposomes. This can be explained by the rapid conversion of LPA into DPPA by the LPAAT enzyme when both proteins are present.

To determine if the lipid products were generated from enzymes co-localizing in the vesicle membrane after co-translational incorporation, or instead, from synthesized enzymes that fail to insert into the lipid bilayer, we carried out experiments where liposomes were omitted during gene expression. When GPAT and LPAAT enzymes were assayed separately in the absence of liposomes, measurable amounts of LPA and DPPA were detected, respectively (Fig 3a). However, the amounts of 16:0 LPA and DPPA formed in one- or two-enzyme reactions were consistently higher by more than one order of magnitude in the presence of vesicles (Fig 3a), indicating that co-translational incorporation of the proteins into a lipid matrix greatly enhances enzymatic activity.

We next investigated the kinetics of 16:0 LPA and DPPA formation in combined gene expression and lipid biosynthesis experiments (Fig 3b–3e). Very few kinetics data are available on the *E*. *coli* GPAT and LPAAT enzymes, all relying on purified proteins or solubilized cell membranes [20,21]. Inside-out proteoliposomes containing either the GPAT or LPAAT protein were produced in the presence of their respective substrates and the enzyme kinetics were monitored (Fig 3b and 3c). The amount of detected 16:0 LPA gradually increased for 4 h at a rate of 2.5 μM/h and subsequently rose abruptly to plateau after about 6 h (Fig 3b). This result suggests that GPAT protein folding or membrane insertion could be the rate-limiting step for product formation in the initial phase of the reaction. The final 42-μM concentration of synthesized LPA corresponds to a consumption of ~40% of palmitoyl-CoA substrate (G3P being present in excess), which we suspect is due to enzyme inhibition by free CoA product [20], protein inactivation or spontaneous cleavage of the palmitoyl-CoA thioesther bond. Moreover, the final amount of 16:0 LPA produced represents around 8% of total lipids forming the vesicles. The LPAAT enzyme converted 16:0 LPA and palmitoyl-CoA into DPPA at an initial rate of 5 μM/h and a maximum concentration of ~21 μM was reached after 15 h (Fig 3c). This final concentration corresponds to ~4% increase in the total amount of phospholipids. The reaction consumed ~40% of the 50 μM of substrates, again suggesting possible reaction inhibition, enzyme inactivation or substrate degradation. The time profiles of LPA and DPPA levels were also analysed by co-expressing both GPAT and LPAAT enzymes in the presence of liposomes along with the G3P and palmitoyl-CoA precursors (Fig 3d and 3e). After a lag phase of approximately 4 h, the concentration of LPA peaked to ~2.3 μM at 8 h and subsequently decreased to equilibrate around 1.5 μM at 16 h (Fig 3d). The amount of accumulated LPA is more than one order of magnitude lower than that with GPAT-only proteoliposomes, which can be attributed to its concurrent consumption by the LPAAT enzyme. The kinetics of DPPA production by LPAAT is initially limited by the rate of LPA formation (Fig 3e). The final concentration of DPPA, ~26 μM, represents a consumption of 52% of palmitoyl-CoA that was initially present at a concentration of 100 μM (two palmitoyl-CoA molecules are consumed to generate one DPPA molecule). This corresponds to ~5% increase in the total amount of phospholipids. Moreover, it is approximately 5 μM more than with LPAAT-only proteoliposomes despite the fact that the IVTT resources and machineries are shared when the two genes are co-expressed. This result suggests enhanced activity when the GPAT and LPAAT proteins work in tandem, underlying the role of the lipid membrane as a functional scaffold. In such a chain reaction the spatial proximity of the enzymes in the lipid matrix may facilitate the transfer of intermediate products from one catalytic site to the other [21]. Alternatively, direct interaction between the GPAT and LPAAT proteins may act as allosteric regulation that enhances mutual activity. Further investigations are needed to validate these hypotheses.

### Enrichment of liposome with synthesized DPPA indicates membrane growth

**Fig 4:**
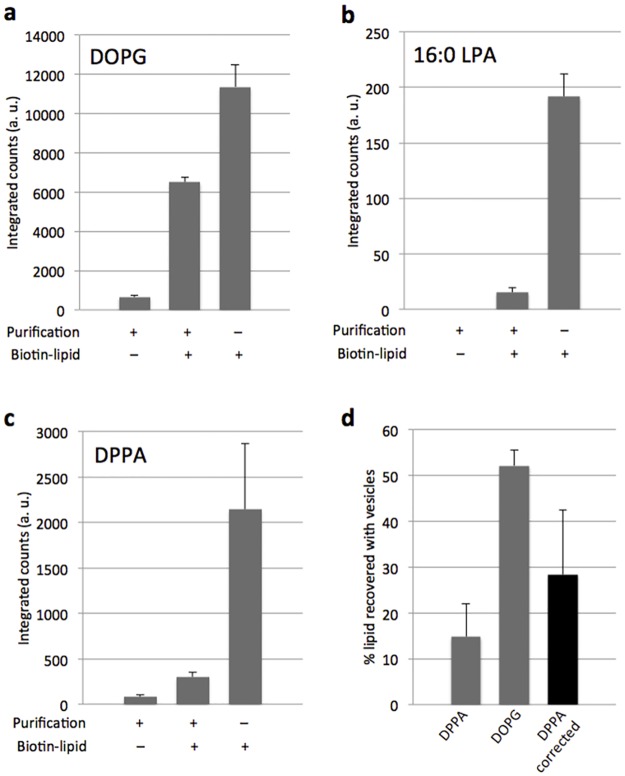
Inside-out acyltransferase proteoliposomes are enriched with synthesized DPPA lipid. (**a–c**) LC-MS analysis of synthesized 16:0 LPA and DPPA lipids with or without liposome purification. Lipid DOPG present in the initial composition of the 400-nm vesicles was used as an internal standard to correct for the loss of lipids during purification. Lipid biosynthesis occurred in a one-pot IVTT and acyl transfer reaction starting from 500 μM G3P and 100 μM p-CoA substrates. In some samples SUV membranes were doped with a biotinylated lipid for immobilization of liposomes on streptavidin-coated magnetic beads. Inspection of the amounts of lipids detected for the different experimental conditions allowed us to discriminate between liposome-integrated and free DPPA. Data are mean and s.e.m. of three independent experiments. For each replicate the same sample was injected two times in the MS, their averaged value was calculated and data are reported as the mean and standard error across the three trials. (**d**) Calculation of the percentage of synthesized DPPA co-localizing with liposome membrane. The use of DOPG as an internal standard enabled the quantification of the fraction of non-immobilized or disrupted vesicles that were washed away during the purification step. Percentage values of recovered DPPA and DOPG were calculated as [*counts*(purif+|biotin+)–*counts*(purif+|biotin–)] / *counts*(purif–|biotin+) × 100. Then, the obtained value for DPPA was divided by that for DOPG to correct for the loss of lipids during purification (Figure C in S1 File), resulting in a value of 28% ± 14% as an estimation of synthesized DPPA that effectively localized in liposomes.

With the ultimate goal to stimulate vesicle growth through phospholipid biosynthesis in mind, we examined where the enzymatically produced DPPA lipid localized. Both GPAT and LPAAT enzymes were co-expressed to form hybrid proteoliposomes and the IVTT system was supplemented with palmitoyl-CoA and G3P precursors to initiate lipid synthesis concurrent to protein production. Liposomes were purified using streptavidin-coated magnetic beads via biotinylated lipids added in the initial membrane composition (Figure C in S1 File). The vesicle content, as represented by DOPG, and the synthesized lipids LPA and DPPA were quantified before and after purification. The fraction of synthesized lipids that co-purified with the vesicles was then calculated (Fig 4). The low number of counts for LPA detected post purification indicates that it does not stably insert into the membrane (Fig 4b). Therefore, it was not possible to accurately determine the LPA membrane fraction after correcting for the loss of lipids during purification and filtering. However, we found that ~28% of *in situ* synthesized DPPA lipids co-purified with liposomes (Fig 4d). This corresponds to a concentration of ~7 μM, which represents an increase of ~1% of the total vesicle surface area (Supplementary text in S1 File).

### *In vesiculo* enzyme production and synthesis of DOPA lipid

**Fig 5:**
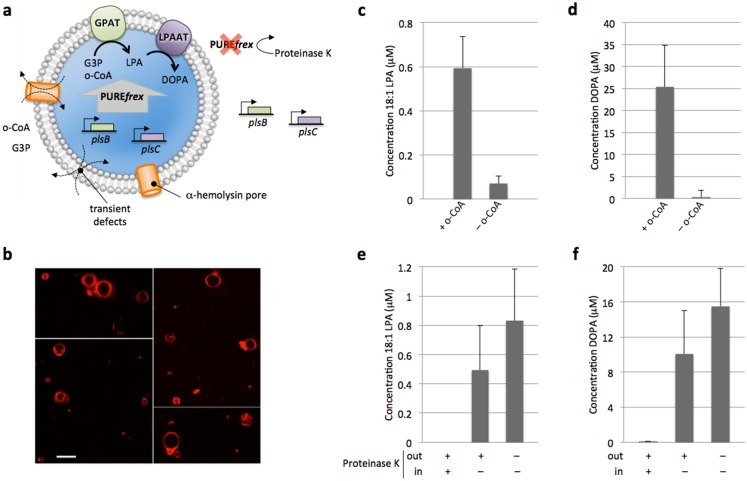
Synthesis of 18:1 LPA and DOPA from GPAT and LPAAT enzymes produced inside liposomes. (**a**) Schematic of vesicle-confined experiments. PURE*frex* supplemented with the *plsB* and *plsC* genes and with 500 μM G3P was encapsulated inside liposomes using gentle rehydration of a lipid film covering sub-millimetre glass beads. Lipid composition consisted of DOPC, DOPE, DOPG, cardiolipin, TexasRed-DHPE and DSPE-PEG-biotin (Table B in S1 File). Swelling occurred at 4°C to avoid reaction initiation. Gene expression outside liposomes was inhibited by protein digestion. Lipid biosynthesis was triggered by external supply of 100 μM oleoyl-CoA (o-CoA). (**b**) Confocal microscopy images of liposomes after swelling. Vesicles were labelled with a membrane dye (Texas-Red). Scale bar is 5 μm. (**c**,**d**) Concentration of 18:1 LPA (**c**) and DOPA (**d**) synthesized in a one-pot reaction by GPAT and LPAAT enzymes produced *outside* liposomes composed of DOPG, DOPE and cardiolipin (Table B in S1 File). Lipid precursors were 500 μM G3P and 100 μM o-CoA (except in negative control). Error bars indicate s.e.m. of two injections of the same sample. (**e**,**f**) Concentration of 18:1 LPA (**e**) and DOPA (**f**) produced by GPAT and LPAAT enzymes generated *inside* liposomes. Three experimental configurations corresponding to different localizations of protein digestion were tested. As expected, addition of Proteinase K both inside and outside liposomes totally inhibited lipid synthesis. In the absence of Proteinase K 18:1 LPA and DOPA accumulated as a result of both internal and external acyltransferase production. Liposome-confined IVTT and lipid synthesis was demonstrated by supplementing Proteinase K outside vesicles according to the reaction scheme illustrated in (a). Data are mean and s.e.m. of three independent experiments. For each replicate the same sample was injected two times in the MS, their averaged value was calculated and data are reported as the mean and standard error across the three trials.

As a next step towards self-producing phospholipid vesicles [22], we used oleoyl-CoA as a substrate to enzymatically produce DOPA lipids whose acyl moieties match that of pre-existing DOPX vesicles (Fig 5). Here, DOPC was removed from the membrane composition to simulate more closely the native *E*. *coli* lipid mixture. Using liposomes consisting of DOPG/DOPE/cardiolipin along with G3P and oleoyl-CoA as substrates, we demonstrated that DOPA, the direct precursor of the vesicle lipids, could be produced by the GPAT and LPAAT enzymes in combined IVTT and acyltransferase activity assays (Fig 5d). Production of the 18:1 LPA intermediate was also detected (Fig 5c), though in lower amount than DOPA due to its subsequent consumption by the LPAAT enzyme. Around 25 μM of DOPA was produced, a concentration similar to that of DPPA when starting from palmitoyl-CoA instead of oleoyl-CoA.

Further, to mimic the cellularization of gene expression, the PURE system together with the *plsB* and *plsC* genes were compartmentalized inside cell-sized liposomes (Fig 5a). These *in vesiculo* experiments aim to recapitulate some essential features specific to the native cellular context, such as confinement and entropy effects, and exposure to lipidic boundaries. Additionally, they may simulate the cytoplasm-like crowding environment in the lumen of the vesicle, as remarkably high concentrations of proteins can be entrapped upon liposome formation [23]. The method to prepare gene expressing-vesicles is based on gentle lipid film swelling and it generates a heterogeneous population of uni- and multilamellar liposomes with sizes ranging from < 0.5 μm to several micrometers in diameter, as visualized on a fluorescence confocal microscope (Fig 5b). Compared to our previously described protocol [24], the complete PURE*frex* system—comprised of the transcription-translation machineries, the tRNAs and the feeding solution—supplemented with G3P and β-mercaptoethanol, was encapsulated inside liposomes. The average number of DNA molecules per 5-μm diameter vesicle is ~30 and ~145 for *plsB* and *plsC* genes, respectively; therefore the DNA copy number per liposome is not a limiting factor [25]. To prevent the reactions from starting prematurely, lipid film swelling was performed at 4°C, which is still above the phase transition temperature of the bilayer. Non-encapsulated proteins were digested by external addition of proteinase K and intravesicular gene expression was simultaneously initiated by raising the temperature to 37°C. In control experiments, proteinase K was added in the swelling medium such that digestion of proteins occurred both inside and outside vesicles. The liposome membrane was equipped with the pore-forming protein α-hemolysin to facilitate the uptake of G3P, amino acids and nucleoside triphosphates present in the external environment, while providing a path for side products removal. After 3 h gene expression, lipid synthesis was triggered by adding the co-substrate oleoyl-CoA from the outside of the vesicles and the solution was incubated overnight at 37°C.

Liposome-confined production of 18:1 LPA and DOPA could clearly be demonstrated (Fig 5e and 5f). As expected, addition of proteinase K completely impedes gene expression and thus lipid synthesis. In the absence of active protein degradation, bulk production of lipids seems to be inefficient since the total amount of DOPA is not largely reduced upon addition of proteinase K despite the relatively large external volume. This result suggests that at least one of the key reaction steps, i.e. gene expression, co-translational membrane insertion, or lipid biosynthesis, is enhanced when compartmentalized inside liposomes. One hypothesis is that *in vesiculo* co-production of GPAT and LPAAT enzymes will give rise to a higher density of the two enzymes in the vesicle membrane, which may favor molecular transfer during the cascade reaction. How the oleoyl-CoA substrate supplied outside the vesicles reaches the GPAT and LPAAT catalytic sites needs clarification. It likely involves a two-step process: the binding to the liposome membrane and flip-flop to reach the enzyme active sites. Because CoA is hydrophilic, the latter step necessarily requires the formation of polar defects in the bilayer to facilitate translocation across the hydrophobic core. Such transient defects might be created by osmotic pressure and possible lipid chain mismatch [24]. Regarding the binding of acyl-CoA to the vesicles, it has already been reported that palmitoyl-CoA preferentially partitions into a phospholipid bilayer [26]. Compared to inside-out proteoliposome experiments, a larger enrichment of the vesicle membrane with DOPA is expected (i.e. > ~28%) when both gene expression and lipid biosynthesis occur inside liposomes.

### Reconstitution of complete phospholipid synthesis pathways for headgroup modifications

**Fig 6:**
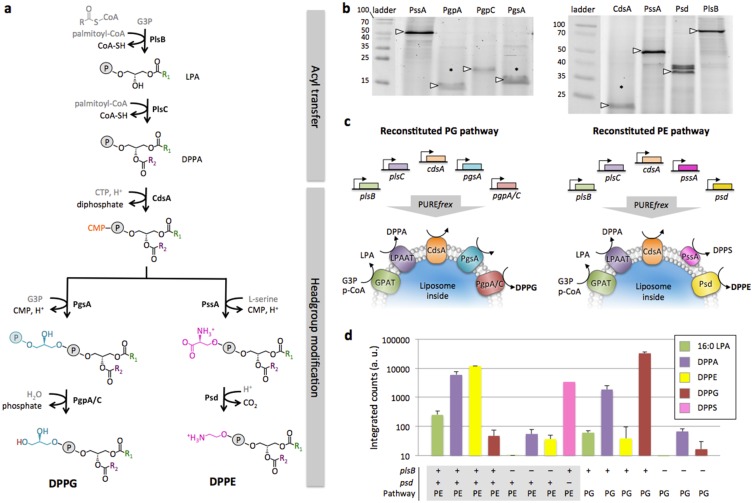
Functional reconstitution of complete biosynthesis pathways for PE and PG lipids. (**a**) Metabolic pathways that lead to the production of DPPG and DPPE starting from palmitoyl-CoA, glycerol-3-phosphate (G3P), cytidine triphosphate (CTP) and L-serine as main substrates. The two-step acyl transfer reaction and the first headgroup conversion step are common to the PE and PG pathways that then branch out into different headgroup modification reactions (see Supplementary text in S1 File for a description of the individual enzymatic steps). For the final step of PG synthesis there exist three alternative enzymes: PgpA, PgpB and PgpC, of which two (A/C) were used in this study. (**b**) Fluorescence scans of SDS-PAGE gels for the headgroup modifying enzymes produced in the PURE system. Fluorescently labeled lysine residues were incorporated during translation. The left gel is 15% polyacrylamide. In addition to the *pssA* gene product that was used as a control, the gene products of *pgpA*, *pgpC* and *pgsA* were synthesized. The right gel is 12% polyacrylamide and, besides the *plsB* gene product used as a control, the genes *cdsA*, *pssA* and *psd* were expressed. Size markers are in kDa. The arrowheads point to the observed protein molecular mass. The symbol “*” indicates the position of the band as expected from the nucleotide sequence of the genes (Supplementary text in S1 File). (**c**) Schematic of the inside-out proteoliposome reconstitution experiments and enzymatic cascade reactions, where all genes of a given pathway were expressed in PURE*frex* and all specific substrates were supplied. (**d**) LC-MS data reporting lipid production in the PE and PG pathways under various experimental conditions. Combined gene expression and lipid biogenesis was carried out as illustrated in (c) using 25 ng of each linear DNA templates, 500 μM G3P, 100 μM palmitoyl-CoA, 1 mM CTP and 500 μM L-serine. Details of MS signatures for the different lipids are reported in Table A in S1 File. Lipids DPPE and DPPG were unambiguously detected in a pathway-specific manner. No PG is produced in the reconstituted PE pathway. Likewise, no PE was detected in the PG pathway. When the *plsB* gene is omitted the complete pathways are shut down. In the absence of the Psd enzyme, PE was not detected and its substrate lipid DPPS accumulated. Note that the MRM data for PS come from the MS optimizer results, not from separate experiments as used for the other compounds. Data are mean and s.e.m. of three independent experiments, except for the negative controls without *plsB* gene where two independent experiments were conducted. For each replicate the same sample was injected between one and four times in the MS, their averaged value was calculated and data are reported as the mean and standard error across the different trials.

Diversity of phospholipid headgroups is crucial for membrane signaling, maintenance of transmembrane potential and activity of membrane proteins [10,27]. Hence, we sought to reconstitute the whole cascade of enzymatic reactions involved to convert PA lipids into the more abundant and bilayer-stable diacyl-PG and diacyl-PE bacterial lipids. The *E*. *coli* PG metabolic pathway relies on the enzymes CdsA, PgsA and Pgp(A, B or C), while the *E*. *coli* PE biosynthesis pathway implicates the CdsA, PssA and Psd enzymes (Fig 6a, Supplementary text). Linear DNA templates encoding each for one enzyme of the cascade were constructed and translation products were verified by gel electrophoresis (Fig 6b). Multiple genes were co-expressed in the PURE system outside preformed liposomes to reconstitute either the PG or PE pathway starting with the GPAT enzyme (Fig 6c). Gene expression and lipid synthesis occurred concurrently with G3P, palmitoyl-CoA, CTP and additionally L-serine for the PE pathway. LC-MS analysis of DPPE and DPPG end products was performed (Fig 6d). We found that DPPG and DPPE phospholipids could successfully be synthesized in a pathway-selective manner. Omitting the gene for the GPAT enzyme shuts the cascade reaction in both metabolic pathways, demonstrating that possible contaminating amounts of 16:0 LPA and DPPA are too low to trigger the chain reactions. Moreover, accumulation of DPPS, the last intermediate product in the PE pathway, could be measured when the *psd* gene was absent (Fig 6d). Cell-free synthesis and reconstitution of the two pathways from the inside of liposomes will necessitate to concatenate all seven genes on a single DNA construct to avoid compositional heterogeneity in the different templates between vesicles and to increase the fraction of liposomes loaded with all genes.

## Discussion

### Reconstitution of membrane-bound enzymatic pathways in the PURE system

Cell-free synthesis of membrane proteins has become instrumental for structural and functional studies of this important class of proteins [28,29]. Tens of different membrane proteins have already been co-translationally reconstituted into liposomes, including the GPAT and LPAAT enzymes studied here [6,28,30,31]. However, the co-reconstitution of even simple enzymatic cascades has remained a challenge. Using the *E*. *coli*-based minimal PURE system as a protein factory and liposomes as functional scaffolds, we have shown here that two entire phospholipid synthesis pathways, comprising five enzymes each for a total of eight different membrane proteins, could successfully be reconstructed. This represents the first example of coupling between membrane metabolic pathway and flow of genetic information *in vitro*.

Our liposome-based cell-free platform is highly versatile in terms of the lipidic composition of the vesicular membrane and orthogonal control of multiple biochemical parameters. Compared to *E*. *coli* extracts, PURE system benefits from remarkably reduced contamination by RNases, proteases and lipids. Besides, in cell extract systems the synthesized proteins are not insulated from endogenous components, which may influence the performance of the reconstituted functions. Because of the reconstituted nature of PURE system with highly specialized enzymes for gene expression, interference between the host protein machinery and the newly synthesized components/functions is limited. At least regarding the membrane proteins studied here, no active membrane translocation complexes are required for insertion into the membrane. We believe that our approach can be adapted to high-content single enzyme studies [32], where the chain of enzymatic reactions could be investigated in thousands of individual liposomes.

### Increasing the yield of synthesized lipids and vesicle growth rate

We identify two remaining barriers that will need to be overcome for substantial volume expansion of a lipidic compartment using the present approach. First, long-acyl chain CoA substrates such as those used in this study are subject to precipitation in the presence of Mg^2+^ [33], which limits their initial concentration (to about 100 μM for palmitoyl-CoA) and, consequently, the final amount of phospholipid produced. Synthesis yield could for instance be improved by using the more soluble substrate acyl-ACP or shorter, more soluble, fatty acid derivatives, provided integration with the fatty acid synthase system [34]. Second, the rate of phospholipid synthesis is only a few micromolar per hour. Increasing the rate of protein production by tuning the concentration of some translation factors, or increasing the number of functionally integrated enzymes in the membrane by reducing the vesicle concentration or improving the fraction of folded proteins may accelerate vesicle growth. Interestingly, *in vesiculo* experiments (Fig 5) show that phospholipid production is more substantive when gene expression and lipid synthesis occurred inside liposomes. This enhanced reaction efficiency prompts us to hypothesize that the vesicle surface area might increase more than 1% as observed in inside-out experiments.

### Expanding the repertoire of cell-free synthesized phospholipids

Capitalizing on the present study, further diversification of lipid structures seems reasonably under reach. Indeed, the enzymatic synthesis of cardiolipin through condensation of two PG, or one PG and one PE lipids, and that of phosphatidylcholine (PC) from CDP-diacylglycerol or PE require only one more enzyme each [10,35,36]. Besides, various precursors, including acyl-ACP, can be used to generate a larger repertoire of lipids that act as topological activators for membrane deformation, as cofactors to assist some protein reactions or as signalling molecules. We also envision the possibility to synthesize novel artificial lipids that would be difficult to generate chemically or *in vivo*. For example fluorescent derivatives of acyl-CoA, such as acyl chain-labeled NBD lipids (NBD, Nitrobenzoxadiazole), might be used as substrates to generate fluorescently labelled phospholipids [37] for real-time monitoring of enzymatic acyl transfer reactions and direct imaging of membrane expansion.

### Gene-based phospholipid synthesis for biogenesis of the compartment of a synthetic minimal cell

Our findings also resonate in the experimental framework of the construction of a minimal cell based on IVTT compartmentalized inside liposomes. As opposed to an approach that would exclusively rely on the use of purified proteins, a construction paradigm based on cell-free gene expression as a protein factory appears more viable. Despite significant advances to endow gene expressing-vesicles with cell-like functionalities [6], many challenging obstacles limit the synthesis and quantitative analysis of a large repertoire of proteins, in particular membrane proteins, which precludes the achievement of elaborate functions, such as compartment growth and division. Here we demonstrated how eight essential enzymes involved in phospholipid biosynthesis, a process of critical importance for cellular growth, could be produced *in vitro* from genomic DNA. Seven different diacyl-glycerophospholipids have been synthesized, namely CDP-diacylglycerol, phosphatidylglycerol phosphate, phosphatidylserine, DPPA, DOPA, DPPE and DPPG. Additionally, a protocol was established to compartmentalize gene expression and lipid synthesis inside cell-sized liposomes. These experiments are foundational to future investigations aiming at reconstituting complete lipid biosynthesis pathways embedded inside the membrane of growing vesicles. Because the production of all necessary enzymes is under transcriptional control the absolute and stoichiometric amounts of the different proteins, and thus of the associated lipids, can precisely be regulated over time, which will be hard to realize using purified proteins only. Such a constructive biology approach, in which the building blocks and processes are directly inspired from those existing in modern organisms, will complement chemical routes using artificial membrane components [38–40] for repetitive growth and fission of liposomal structures.

### Cell-free lipid biosynthesis as a route for division of a reconstituted minimal cell

Besides volume expansion, we postulate that *in vesiculo* lipid biosynthesis could be exploited to change the equilibrium state of the membrane and triggers asymmetric division [41]. First, in light of the recently unveiled mechanism of L form cell reproduction [42], we predict that internal synthesis of phospholipids could be sufficient to induce shape deformation as a manifestation of the excess surface area of the membrane. The resulting unbalanced surface-to-volume ratio will eventually be released by division through budding. Second, when coupled to excess membrane generated by lipid production or by crossing the bilayer phase transition temperature [43,44], the synthesis of topologically active lipids (e.g. PE) could stimulate shape transformation and complete fission of vesicles.

## Materials and Methods

### Materials

Palmitoyl coenzyme A (p-CoA), oleoyl coenzyme A (o-CoA), palmitoyl lysophosphatidic acid (16:0 LPA), oleoyl lysophosphatidic acid (18:1 LPA), 1,2-dipalmitoyl-*sn*-glycero-3-phosphate (DPPA), 1,2-dioleoyl-*sn*-glycero-3-phosphate (DOPA), 1,2-dioleoyl-*sn*-glycero-3-phosphocholine (DOPC), 1,2-dioleoyl-*sn*-glycero-3-phosphatidylethanolamine (DOPE), 1,2-dioleoyl-*sn*-glycero-3-phospho-(1'-*rac*-glycerol) (DOPG), 1',3'-bis[1,2-dioleoyl-*sn*-glycero-3-phospho]-*sn*-glycerol (cardiolipin), and 1,2-distearoyl-*sn*-glycero-3-phosphoethanolamine-N-[biotinyl(polyethylene glycol)-2000] (DSPE-PEG-biotin) were purchased from Avanti Polar Lipids. N-(6-tetramethylrhodaminethiocarbamoyl)-1,2-dihexadecanoyl-*sn*-glycero-3-phosphoethanolamine (TRITC-DHPE) was from Invitrogen. Texas Red 1,2-dihexadecanoyl-*sn*-glycero-3-phosphoethanolamine, triethylammonium salt (Texas Red DHPE), 212 μm-300 μm acid washed glass beads, chloroform, methanol, acetylacetone, glycerol-3-phosphate (G3P), β-mercaptoethanol, and L-serine were from Sigma-Aldrich. Formic acid, ammonium formate and ULC grade organic solvents for mobile phases were from Biosolve. Cytidine triphosphate (CTP) was from Promega.

### Buffers

Buffer A (GPAT buffer, 150 mM Tris-HCl, 400 mM NaCl, 3 mM MgCl_2_, 5 mM β-mercaptoethanol, 1 mg/mL BSA, pH 8.4), buffer B (LPAAT buffer, 100 mM Tris-HCl, 200 mM NaCl, 0.5 mM MgCl_2_, 1 mg/mL BSA, pH 9.0), buffer C (50 mM HEPES, 100 mM potassium glutamate, 13 mM magnesium acetate, pH 7.6), buffer D (20 mM HEPES, 180 mM potassium glutamate, 14 mM magnesium acetate, pH 7.6), buffer E (GPAT dialysis buffer, 150 mM Tris-HCl, 400 mM NaCl, 3 mM MgCl_2_, pH 8.4), buffer F (LPAAT dialysis buffer, 100 mM Tris-HCl, 200 mM NaCl, 0.5 mM MgCl_2_, pH 9.0), buffer G (150 mM Tris-HCl, 400 mM NaCl, 3 mM MgCl_2_, 1 mg/ml BSA, 66.6 μM G3P, pH 8.4).

### Preparation of DNA constructs

The genes *plsB* and *plsC* were kindly provided by Dr. Yutetsu Kuruma [15] in the form of circular plasmids. The plasmids carrying an ampicillin selection marker were amplified in *E*. *coli* (TOP10) cells and purified with a PureYield^™^ plasmid miniprep (Promega). Linear DNA templates were generated by PCR using the primers reported in Table C in S1 File. For the lipid headgroup modification experiments the DNA templates containing the genes *cdsA*, *pgsA*, *pgpA*, *pgpC*, *psd* or *pssA* were all constructed from *E*. *coli* MG1655 (K12) genomic DNA that was extracted with GenElute^™^ Bacterial Genomic DNA Kit (Sigma Aldrich). The genes were cloned into a pET11a backbone by Gibson assembly [45,46]. Amplification of the target genes was performed using two primers. A forward primer with the following regions (from 5’ to 3’): a region overlapping the pET11a sequence and a region overlapping the gene of interest. A reverse primer with the same sequence design (pET11a homology, gene of interest homology) was employed. The assembled plasmids were then amplified in *E coli* cells (TOP10), purified and linear PCR products were generated as described above. All sequences were confirmed by sequencing. The list of primers is reported in Table C in S1 File. Proteins were then synthesized *in vitro* using either the plasmid or linear DNA as a template for gene expression.

### Preparation of small unilamellar vesicles (SUVs)

Lipids dissolved in chloroform were transferred to a 2-mL glass vial. Unless otherwise indicated the regular lipid composition was DOPC/DOPE/DOPG/cardiolipin, 50.8:35.6:11.5:2.1 in mol. %. An overview of the different lipid mixtures used is provided in Table B in S1 File. Chloroform was evaporated under gentle argon flow. Traces of solvent were removed by placing the lipid film-containing vial in a vacuum desiccator for 1 h. The lipid film was then hydrated with buffer D (Figs 2a and 3–6) or buffer C (Figs 1b and 2b). The sample was vortexed to resuspend the lipids in aqueous solution and the produced multilamellar liposomes were subjected to five freeze-thaw cycles in liquid nitrogen, except in proteoliposome purification by flotation (Fig 1b) and CoA assay experiments (Fig 2b), where this step was omitted. Next, the liposomes were extruded 20 times through a polycarbonate membrane with 0.4 μm (Figs 2a and 3–6) or 0.1 μm (Figs 1b and 2b) pores using an Avanti mini-extruder (Avanti Polar Lipids). Finally, the SUV samples were aliquoted, snap-frozen in liquid nitrogen and stored at –20°C until use.

### Cell-free protein synthesis

*In vitro* transcription-translation (IVTT) reactions were performed in the PURE*frex* kit (GeneFrontier Corporation, Japan). PURE*frex* is composed of three different solutions: the enzyme mixture (T7 RNA polymerase, translation factors, energy recycling system, etc.), the *E*. *coli* ribosomes, and the feeding mixture (amino acids, NTPs, tRNAs, creatine phosphate, etc.). All solutions were aliquoted in small volumes and stored at –80°C. For bulk experiments, in which IVTT occurred outside or in the absence of liposome, the PURE*frex* reaction solution was assembled on ice by mixing one volume equivalent (equiv.) of enzyme mix, 1 equiv. of ribosome, 10 equiv. of feeding mix, the DNA template(s) (final concentration ranging from 1.7 nM to 16.9 nM, and the total volume was adjusted to 20 equiv. with nuclease-free water. Both purified plasmids and PCR products have been used as templates. Less truncated transcription products and higher yield of full-length protein were observed when using the *plsB* plasmid and the *plsC* linear DNA (not shown); these conditions were therefore chosen for all reported IVTT reactions. Total reaction volumes of 2.5 μL to 20 μL were used. When indicated, Superase RNase inhibitor (Thermo Fisher), β-mercaptoethanol, lipid precursors and SUVs were supplemented to IVTT reactions (see below). For SDS-PAGE analysis of the translation products, ~5% v/v of stock solution of BODIPY-Lys-tRNA_Lys_ (FluoroTect^™^ GreenLys, Promega), a fluorescence-based *in vitro* translation labeling system, was included to the reaction. Gene expression reactions were carried out at 37°C for 3 h unless coupled with *in situ* lipid biosynthesis.

### Sequential IVTT and acyl transfer reactions

Enzyme-containing proteoliposomes were prepared by performing PURE*frex* reactions using 10 ng/μL of *plsB* and/or *plsC* DNA templates, 0.4 U/μL of RNase inhibitor and 400-nm SUVs (2 g/L lipid). The DHFR-encoding expression plasmid provided in the PURE*frex* kit (5.78 ng/μL final) was used in control experiments for GPAT activity. A linear DNA coding for the LacI protein (20.8 ng/μL final) was employed in control experiments for LPAAT activity. Lipid substrate palmitoyl-CoA was dissolved in a solvent mixture chloroform:methanol:water with vol. % 80:20:2 and 16:0 LPA was dissolved in chloroform:methanol:water with vol. % 65:35:8. The solutions were transferred to a 1.5-mL glass vial and the solvent was evaporated at room temperature and ambient pressure under a chemical hood. The dried lipid substrates were then resuspended in a pre-ran proteoliposome-containing IVTT solution that was diluted ten times either in buffer A (GPAT buffer) or buffer B (LPAAT buffer). The final concentration of palmitoyl-CoA was either 133.3 μM when both GPAT and LPAAT enzymes were co-assayed or 66.6 μM for single-enzyme assays. In addition, reactions with only the LPAAT (or LacI) enzyme included 66.6 μM LPA. All reactions with the GPAT (or DHFR) protein contained 66.6 μM G3P. Negative controls for reactions with both GPAT and LPAAT proteins were performed without G3P. The samples were incubated overnight at 22°C and assayed by LC-MS.

### Acyl transfer fluorescence-based assay

Using acyl-CoA as the acyl donor substrate for GPAT and LPAAT leads to the release of CoA. Accumulation of free CoA was measured by using an acyltransferase activity kit (Enzo Life Sciences), in which CoA reacts with a fluorogenic substrate to form a fluorescent thiol adduct. The acyl transfer activity of the GPAT and LPAAT enzymes was assayed subsequently to protein synthesis and liposome reconstitution in PURE*frex*. Conditions for IVTT reactions in 10-μL volume were as described above with the following modifications: 100-nm SUVs were used at a concentration of 2 g/L and the RNase inhibitor was at 0.2U/μL. The DHFR-encoding plasmid (5.78 ng/μL final) provided as a positive expression template in the PURE*frex* kit and a linear DNA coding for the LacI protein (20.8 ng/μL final) were used as DNA templates in negative control experiments for GPAT and LPAAT activity, respectively.

Because the CoA-sensitive assay is not compatible with the presence of reducing agents, the DTT contained in PURE*frex* was dialyzed out overnight at 4°C using a floating dialysis membrane (V-Series from Millipore) with 25-nm pore size on 100 mL of buffer E (GPAT dialysis buffer) or buffer F (LPAAT dialysis buffer). The 10-μL dialyzed samples were diluted to 100 μL to reach final composition of buffer G for GPAT or buffer B for LPAAT. The solutions were then used to re-suspend the lipid substrates dried into glass vials. Final concentrations were 66.6 μM of palmitoyl-CoA for GPAT activity assay, and 33.3 μM of palmitoyl-CoA along with 33.3 μM of LPA for LPAAT activity assay. The samples were incubated for 2 h at room temperature and assayed according to the instructions of the supplier. Briefly, 25 μL of sample was mixed with 25 μL of proprietary “Transferase Assay Buffer”. The samples were further mixed with 50 μL of ice-cold isopropanol, then with 100 μL of detection solution and incubated 10 min. Fluorescence signal was measured with a CLARIOstar (BMG LABTECH, Germany) or Cary Eclipse (Agilent Technologies) plate reader (results were combined by normalizing to positive control) at 486 nm excitation and 540 nm emission wavelengths.

### Combined IVTT and acyl transfer reactions

PURE*frex* solutions were assembled using 3.4 nM *plsB* and 16.9 nM *plsC* DNA templates supplemented with 0.4 g/L of 400-nm SUVs, 0.4 U/μL of RNase inhibitor, 5 mM β-mercaptoethanol and 500 μM G3P. Lipid precursors palmitoyl-CoA and oleoyl-CoA were separately dissolved in a solvent mixture chloroform:methanol:water with vol. % 80:20:2 and 16:0 LPA in chloroform:methanol:water with vol. % 65:35:8. The solutions were transferred to a 0.2-mL Eppendorf PCR tube and the solvent was evaporated at room temperature and ambient pressure under a chemical hood. The dried lipid substrates were then resuspended in the PURE*frex* mix leading to the final concentrations of 100 μM palmitoyl-CoA or oleoyl-CoA, and 100 μM 16:0 LPA, with the exception of the kinetics experiments (Fig 3b–3e), where 50 μM palmitoyl-CoA and 50 μM 16:0 LPA were used with LPAAT-only. The samples were incubated at 37°C overnight or shorter when indicated, and the lipid content was assayed by LC-MS.

### Purification of proteoliposomes by floatation

A 20-μL IVTT reaction was run with 12.5 ng/μL of the *plsB* and/or *plsC* constructs and 1.66 g/L of 100-nm SUVs. The reaction solution was further supplemented with 0.8 μL of BODIPY-Lys-tRNA_Lys_. The *in vitro* synthesized acyltransferase GPAT and LPAAT enzymes successfully reconstituted in proteoliposomes were separated from bulk proteins, including all PURE*frex* components, by liposome floatation technique. First, the pre-ran IVTT reaction solution was mixed with 1 μL of RQ1 DNase and 1 μL of RNase I, and held at 37°C for 1 h. The sample was then diluted two times in buffer C, layered on top of 40 μL of a 7.5% w/v sucrose solution in a 230-μL ultracentrifugation tube (Beckmann Coulter). The sample was spun at 40,000 rpm (203,000 g) for 4 h using a 42.2 Ti rotor in an Ultima L-90K centrifuge (Beckmann Coulter). The floating liposomes—labelled with the DHPE-TRITC membrane dye for easy visualization—were then harvested with a cut pipette tip from the surface and the co-purified proteins were analysed by SDS-PAGE.

### SDS-PAGE analysis

For SDS-PAGE analysis of protein synthesis, samples containing the GreenLys-labelled translation products were denatured for 2 min at 65°C, loaded on a 12% or 15% SDS-PAGE gel and analyzed using a fluorescence gel imager (Typhoon, Amersham Biosciences). Ladder was either prestained or stained with coomassie blue and appended to the images to scale with GIMP image editor.

### Liposome purification with Dynabeads^®^

For the experiments of LC-MS analysis of liposome enrichment with synthesized lipids, PURE*frex* solutions were assembled with 10 ng/μL of *plsB* and *plsC* templates, 0.5 U/μL RNase inhibitor, 5 mM β-mercaptoethanol, 500 μM G3P and 2 μg/μL 400-nm SUVs with (two samples pooled) or without (one sample) biotinylated lipids (lipid mix contained 0.1% DSPE-PEG biotin). The three PURE*frex* solutions were added separately to 100 μM of dried palmitoyl-CoA and reacted overnight at 37°C. Two samples, one with biotinylated liposomes and the other with non-biotinylated liposomes, were subjected to purification using streptavidin-coated magnetic beads. The overall sample purification workflow is illustrated in Figure C in S1 File. Thirty microliter of M-270 Dynabeads^®^ (Life Technologies Europe BV) were washed three times with 200 μL of buffer D. The beads were resuspended in 100 μL of buffer D and 12.5 μL of the proteoliposome-containing solutions was added to separate tubes. The samples were then incubated at room temperature in a rotator for 80 min. The beads were then washed five times with 200 μL of buffer D and the sample volume was adjusted to 12.5 μL. Each of the three 12.5-μL samples (two undertook purification with Dynabeads^®^, one did not) was mixed with 387.5 μL methanol and then sonicated for 10 min. The samples were then supplemented with 1.6 mL methanol (final volume 2 mL) and filtered using a 0.2-μm Acrodisc^®^ (Pall) syringe filter to remove the magnetic beads. The volume of the solution was finally reduced to approximately 112.5 μL with an Eppendorf Concentrator Plus and 12.5 μL of MilliQ water was added along with 1.25 μL of 500 mM EDTA and 1.25 μL of 200 mM (equivalent to 20.6% v/v) acetylacetone. The samples were then assayed by LC-MS.

### *In vesiculo* gene expression and acyl transfer experiments

Liposomes were formed by natural swelling of a lipid film coated onto 212–300-μm glass beads according to our previously reported protocol [24], with some modifications. Five milligrams of lipids dissolved in chloroform were mixed in a round-bottom glass flask. Lipid composition was DOPC, DOPE, DOPG and cardiolipin at 50.8:35.6:11.5:2.1 mol. %. The mixture was supplemented with TexasRed-DHPE 0.5% and DSPE-PEG-biotin 1%, both in mass percent. To improve lipid film swelling at low temperature, ~63.5 μmol. of rhamnose (from a 100-mM stock solution in methanol) was added to the lipid mixture [47]. Finally, 1.5 g of glass beads was added and the organic solvent was rotary evaporated at 200 mbar at room temperature. The dried lipid-coated beads were transferred to a 2-mL polypropylene tube and put in a vacuum dessicator overnight to remove traces of solvent. The beads were then stored under argon at –20°C and were re-dessicated for 30 min before use.

The IVTT reaction solution to be internalized inside liposomes was prepared by pooling the three PURE*frex* reagents in a ratio feeding/ribosome/enzymes of 10:1:1 supplemented with 7.4 ng/μL of *plsB* and *plsC* DNA templates (final concentrations 2.5 nM and 12.5 nM, respectively), 0.74 U/μL RNase inhibitor, 7.4 mM β-mercaptoethanol and 740 μM G3P. The solution was split into three samples of 13.5 μL each. To one sample 1 μL of 100 μg/mL proteinase K (from Promega, stock solution in MilliQ water) was added, while in the other two samples 1 μL of MilliQ was injected. The IVTT mixture was then added to lipid-coated glass beads to form liposomes. Lipid film swelling was performed for 2 h at 4°C to avoid gene expression to start, while maintaining the bilayer in the liquid disordered phase. After swelling the samples were subjected to four freeze-thaw cycles (alternating exposure to 4°C and liquid nitrogen) to break multilamellar structures. From each reaction 4.35 μL of supernatant was harvested, mixed with 1.35 μL of 4.44 μM α-hemolysin (Sigma-Aldrich) and incubated at room temperature for 10 min. To one of the samples that have not received proteinase K, 0.3 μL of the protease stock solution was added to digest all proteins outside liposomes, while 0.3 μL of MilliQ was added in the other two samples. All three samples were then incubated at 37°C for 3 h. The 6-μL samples were transferred into new 0.2-mL PCR tubes, where dried oleoyl-CoA had been deposited, leading to a final concentration of lipid precursor of 100 μM. The samples were further incubated at 37°C overnight and their lipid contents were assayed by LC-MS.

### Headgroup modification experiments

Cell-free gene expression, acyl transfer and headgroup conversion reactions were performed in a single-pot reaction in an inside-out proteoliposome configuration essentially as described in the section ‘Combined IVTT and acyl transfer reactions’. Here, five different DNA templates (each 5 ng/μL) each encoding for one enzyme of the PE or PG pathways were included in 5-μL PURE*frex* solutions to reconstitute the whole cascade of enzymatic reactions, except for control experiments where the *plsB* or *psd* gene was omitted. The lipid precursor palmitoyl-CoA was used at a final concentration of 100 μM to produce the DPPE and DPPG output lipids. In addition, 1 mM CTP and 500 μM L-serine were included as cofactors in all reactions for synthesis of DPPE and DPPG.

### Liquid chromatography-mass spectrometry (LC-MS) for lipid detection

The lipid fraction was extracted by first diluting the samples ten times with methanol containing 2 mM (0.0206% vol.) acetylacetone. For *in vesiculo* and headgroup conversion experiments 5 mM of EDTA was also included in the organic solvent to improve the stability of the LC-MS method (Figure D in S1 File). The samples were then sonicated for 10 min in a bath sonicator, spun down at 16,100 r.c.f. in an Eppendorf 5415R to remove protein and nucleic acid precipitates, and the supernatant was harvested for LC-MS analysis.

The LC-MS method for lipid analysis was adapted from previous studies [48,49]. In all experiments, 5-μL samples were injected into an LC system (Agilent 1260) equipped with a XSELECT HSS T3 2.5 μm analytical column. Lipids elute sequentially (a typical chromatogram is shown in Fig 1c) providing a pre-separation step before entering the MS system. The mobile phase A consisted of (all % given in vol./vol.) 60% acetonitrile, 40% deionized water, 7 mM ammonium formate, 0.0114% formic acid and 2 mM acetylacetone. Formic acid and ammonium formate were used to set the pH to 4.0. The mobile phase B was 90% isopropanol, 10% acetonitrile, 0.0378% formic acid and 2 mM acetylacetone. Acetylacetone was used to chelate metal ions, which reduced peak tailing of LPA and DPPA [50].

The output from the analytical column was connected to an MS instrument (Agilent 6460 Triple Quad MS) that was operated according to the parameters reported in Table A in S1 File, where the values in non-calibrated data represent the integrated peak area. We adopted a multiple reaction monitoring (MRM) transitions analysis, whereby the lipids were ionized, selected by mass in a first ion filter, fragmented by collisions with nitrogen gas in a collision cell and then the fragment masses were monitored in another ion filter.

### LC-MS data calibration curves

When indicated (Figs 3b–3e and 5), the absolute amounts of synthesized lipids were determined using purified lipids of known concentrations to plot calibration curves (Figure B in S1 File). The full method is described in the Supplementary text.

### Fluorescence confocal microscopy

Liposome samples were prepared according to the protocol used for vesicle-confined reactions. After lipid film swelling, a 6-μL-solution droplet was deposited into a homemade chamber mounted onto a #1.5 glass coverslip. The sample was imaged using a fluorescence confocal microscope (A1^+^ from Nikon) equipped with a ×100 oil immersion objective and a 561-nm laser line with appropriate dichroic mirror and emission filter to detect the TexasRed-DHPE membrane dye.

### Statistical analysis of data

Whenever indicated a two-sample *t*-test (unequal variances) was performed using MATLAB (MathWorks), with the hypothesis that the population means are unequal.

## Supporting Information

S1 File Supporting information file contains supplementary Tables, Figures and text. Supplementary text includes details on (i) lipid handling, (ii) the preparation of lipid standards and calibration curves for absolute quantitation of synthesized lipids, (iii) the calculation of vesicle surface area increase and number of membrane proteins per liposome, and (iv) a description of the main structural and reactivity properties of the phospholipid headgroup modifying enzymes. Table A in S1 File. Optimized parameters for mass spectrometry Multiple Reaction Monitoring (MRM) mode.*The fragmentor voltage was set to 220 mV with PgpA and to 70 mV with PgpC. **Table B in S1 File. Overview of the different lipid compositions used. Table C in S1 File. List of primers and sequences used for Gibson assembly or for generating linear DNA templates for IVTT reactions.** The lower case three-nucleotide stretch in the DNA sequences denotes the region in the primer where the coding region starts. All sequences are reported in the 5’ to 3’ direction. **Figure A in S1 File. Chromatogram profiles of the lipids used in this study.** Chromatogram profiles of purified lipids as measured with LC-MS. Lipids DPPA and DOPA were identified using three different fragment ions to facilitate unambiguous detection. Their corresponding m/z of intact and fragment ions are appended in the chromatograms. **Figure B in S1 File. Calibration curves for quantifying absolute lipid concentrations.** The number of counts for lipid standard samples of known concentrations was plotted against the concentration and a linear fit of the data was performed. For each sample, the concentration error was estimated to be 10% based on the fact that lipids obtained from Avanti Polar Lipids are overpacked by as much as 10% and that standard stocks may experience degradation. The counts error was calculated directly from multiple measurements of lipid standards. Alternatively the error percentage was calculated from multiple injections of synthesized lipids-containing samples as an estimate of the MS data variability on a given day. The concentration to counts conversion factors are calculated from the slopes divided by ten to account for the ten-fold dilution of samples before injection to the MS. (**a**) Calibration plots for the kinetics measurements presented in Fig 3b–3e. Values of conversion factors are 1302 cts/μM for LPA and 778 cts/μM for DPPA. (**b**) Calibration plots for the oleoyl-CoA bulk experiments presented in Fig 5c and 5d. Values of conversion factors are 770 cts/μM for 18:1 LPA and 393 cts/μM for DOPA. (**c**) Calibration plots for the oleoyl-CoA *in vesiculo* experiments presented in Fig 5e and 5f. Values of conversion factors are 531 cts/μM for 18:1 LPA and 604 cts/μM for DOPA. Calibration curves were performed on the same day as measurements on stored samples to minimize differences observed when experiments were made on different days. This, together with contribution from the fact that preparation of lipid standards was different for the three sets of experiments (Supplementary text), explains the different conversion factor values obtained in b) and c). **Figure C in S1 File. Experimental workflow for assessing the fraction of synthesized DPPA localized in the liposome membrane.** The loss of lipids during the filtration step was determined by measuring the number of counts of lipid standards (DPPA and DOPG, three concentrations each) treated with or without filtration. Linearity over the used concentration range was validated for both lipids and both treatments (not shown). For each lipid the loss introduced by filtering was calculated as: Loss % = slope _filtered_ / slope _unfiltered_ x 100. Values of 42% and 55% were obtained for DOPG and DPPA, respectively. The fraction of DOPG and DPPA lipid retained during purification was assessed using biotin-labelled vesicles and subjecting, or not, samples to purification with Dynabeads. Recovered lipid values correspond to 57% and 14% for DOPG and DPPA, respectively. The sample devoid of biotinylated lipid served to infer the loss of DPPA due to nonspecific adsorption to the magnetic beads or to the tube during purification. The corresponding count number was subtracted from that of the biotin-labelled vesicle sample to determine the actual fraction of DPPA that was retained through liposome immobilization. In Fig 4, we estimated this fraction to represent ~15% of the total DPPA synthesized, which after correcting for the fact that only ~52% of internal standard DOPG is recovered leads to ~30%. **Figure D in S1 File. Addition of EDTA in MS samples leads to higher number of counts for DPPA.** A higher number of counts for DPPA was observed when standard solutions were supplemented with 5 mM EDTA. These results suggest that interaction between the charged phosphate group of the lipids, metal ions and exposed silica in the column could lower the absolute number of counts. We therefore included EDTA in some samples (*in vesiculo*, headgroup modification and Dynabeads purification experiments) to increase signal and reproducibility. Standard sample matrix corresponds to 90% methanol with 2 mM acetylacetone and 10% buffer D. **Figure E in S1 File. Efficiency of protein separation by the liposome floatation technique as measured by SDS PAGE.** PURE*frex* reactions were carried out as described in the Methods section. Production of the LacI and DHFR proteins was used as a control for removal of non-membrane-bound proteins. The same SDS PAGE was analysed through fluorescence of nonnatural amino acids as a marker of translation products (top image) and by CBB staining as a total protein marker (bottom image). Lane 1, V849a protein ladder; Lane 2, no DNA non-purified; Lane 3, LacI non-purified; Lane 4, DHFR non-purified; Lane 5, LacI purified, bottom ¼; Lane 6, Lac I purified, second ¼; Lane 7, LacI purified, third ¼; Lane 8, LacI purified, top ¼. Arrowheads indicate the synthesized LacI protein in the unpurified and bottom ½ fractions of the purified samples. Good removal of bulk protein is achieved by harvesting the top ½ of the sample, as can be seen from the protein-free lanes 7 and 8. Hence, isolation of membrane-bound GPAT and LPAAT proteins that co-purified with liposomes was ensured by harvesting a sample fraction < ¼ from the top (Fig 1b). This experiment also shows that water-soluble proteins containing fluorescent amino acids do not significantly bind to the vesicle membrane in a non-specific manner.
(PDF)

## References

[1] Luisi PL. Toward the engineering of minimal living cells. The Anatomical Record 2002;268: 208–214. 10.1002/ar.10155 12382319

[2] Luisi PL, Ferri F, Stano P. Approaches to semi-synthetic minimal cells: a review. Naturwissenschaften 2006;93: 1–13. 10.1007/s00114-005-0056-z 16292523

[3] Forster AC, Church GM. Towards synthesis of a minimal cell. Molecular Systems Biology 2006;2: 45 10.1038/msb4100090 16924266 PMC1681520

[4] Noireaux V, Maeda YT, Libchaber A. Development of an artificial cell, from self-organization to computation and self-reproduction. Proceedings of the National Academy of Sciences 2011;108: 3473–3480. 10.1073/pnas.1017075108 21317359 PMC3048108

[5] Schwille P. Bottom-up synthetic biology: engineering in a tinkerers world. Science 2011;333: 1252–1254. 10.1126/science.1211701 21885774

[6] Caschera F, Noireaux V. Integration of biological parts toward the synthesis of a minimal cell. Current Opinion in Chemical Biology 2014;22: 85–91. 10.1016/j.cbpa.2014.09.028 25285755

[7] Cronan JE. Bacterial Membrane Lipids: Where Do We Stand? Annual Review of Microbiology 2003;57: 203–224. 10.1146/annurev.micro.57.030502.090851 14527277

[8] Yao J, Rock CO. Phosphatidic acid synthesis in bacteria. Biochimica et Biophysica Acta (BBA)-Molecular and Cell Biology of Lipids 2013;1831: 495–502. 10.1016/j.bbalip.2012.08.018 22981714 PMC3548993

[9] Röttig A, Steinbüchel A. Acyltransferases in bacteria. Microbiology and Molecular Biology Reviews 2013;77: 277–321. 10.1128/MMBR.00010-13 23699259 PMC3668668

[10] Sohlenkamp C, Geiger O. Bacterial membrane lipids: diversity in structures and pathways. FEMS Microbiology Reviews 2015; fuv008. 10.1093/femsre/fuv008 25862689

[11] Dowhan W. A retrospective: use of *escherichia coli* as a vehicle to study phospholipid synthesis and function. Biochimica et Biophysica Acta (BBA)-Molecular and Cell Biology of Lipids 2013;1831: 471–494. 10.1016/j.bbalip.2012.08.007 22925633 PMC3513495

[12] Deamer DW, Gavino V. Lysophosphatidylcholine acyltransferase: purification and applications in membrane studies. Biomembranes and Cell Function 1983;414: 90–96. 10.1111/j.1749-6632.1983.tb31677.x 6584078

[13] Schmidli PK, Schurtenberger P, Luisi PL. Liposome-mediated enzymatic synthesis of phosphatidylcholine as an approach to self-replicating liposomes. J. Am. Chem. Soc. 1991;113: 8127–8130. 10.1021/ja00021a043

[14] Wick R, Luisi PL. Enzyme-containing liposomes can endogenously produce membrane-constituting lipids. Chemistry and Biology 1996;3: 277–285. 10.1016/S1074-5521(96)90107-6 8807855

[15] Kuruma Y, Stano P, Ueda T, Luisi PL. A synthetic biology approach to the construction of membrane proteins in a semi-synthetic minimal cells. Biochimica et Biophysica Acta (BBA) Biomembranes 2009;1788: 567–574. 10.1016/j.bbamem.2008.10.017 19027713

[16] Shimizu Y, Inoue A, Tomari Y, Suzuki T, Yokogawa T, Nishikawa K, et al Cell-free translation reconstituted with purified components. Nat. Biotechnol. 2001;19: 751–755. 10.1038/90802 11479568

[17] Larson TJ, Lightner VA, Green PR, Modrich P, Bell RM. Membrane phospholipid synthesis in Escherichia coli. Identification of the sn-glycerol-3-phosphate acyltransferase polypeptide as the plsB gene product. Journal of Biological Chemistry 1980;255: 9421–9426. 6997313

[18] Coleman J. Characterization of the Escherichia Coli gene for 1 acyl-sn-glycerol-3-phosphate acyltransferase (plsC). Molecular and General Genetics 1992;232: 295–303. 1557036 10.1007/BF00280009

[19] Wilkison WO, Walsh JP, Corless JM, Bell RM. Crystalline Arrays of the Escherichia Coli sn-Glycerol-3-phosphate acyltransferase, an Integral Membrane Protein. The Journal of Biological Chemistry 1986;251: 9951–9968. 3525537

[20] Green PR, Merrill AH, Bell RM. Membrane phospholipid synthesis in Escherichia Coli. Purification, reconstitution, and characterization of sn-glycerol-3-phosphate acyltransferase. The Journal of Biological Chemistry 1981;256: 11151–11159. 7026564

[21] Kessels J, Ousen H, Van den Bosch H. Facilitated utilization of endogenously synthesized lysophosphatidic acid by 1-acylglycerophosphate acyltransferase from escherichia coli. Biochimica et Biophysica Acta (BBA)-Lipids and Lipid Metabolism 1983;753: 227–235. 10.1016/0005-2760(83)90011-5 6351928

[22] Varela FG, Maturana HR, Uribe R. Autopoiesis: the organization of living systems, its characterization and a model. Biosystems 1974;5: 187–196. 10.1016/0303-2647(74)90031-8 4407425

[23] Pereira de Souza T, Steiniger F, Stano P, Fahr A, Luisi PL. Spontaneous crowding of ribosomes and proteins inside vesicles: a possible mechanism for the origin of cell metabolism. ChemBioChem 2011;12: 2325–2330. 10.1002/cbic.201100306 21830290

[24] Nourian Z, Roelofsen W, Danelon C. Triggered gene expression in fed-vesicle microreactors with a multifunctional membrane. Angewandte Chemie 2012;51: 3114–3118. 10.1002/anie.201107123 22246637

[25] Nourian Z, Danelon C. Linking genotype and phenotype in protein synthesizing liposomes with external supply of resources. ACS Synthetic Biology 2013;2: 186–193. 10.1021/sb300125z 23656477

[26] Requero MA, Goñi FM, Alonso A. The membrane-perturbing properties of palmitoyl-coenzyme A and palmitoylcarnitine. A comparative study. Biochemistry 1995; 34(33):10400–10405. 10.1021/bi00033a011 7654694

[27] Zhang Y-M, Rock CO. Membrane lipid homeostasis in bacteria. Nature Reviews Microbiology 2008;6: 222–233. 10.1038/nrmicro1839 18264115

[28] Junge F, Haberstock S, Roos C, Stefer S, Proverbio D, Dötsch V, et al Advances in cell-free protein synthesis for the functional and structural analysis of membrane proteins. New Biotechnology 2011;28: 262–271. 10.1016/j.nbt.2010.07.002 20637904

[29] Sachse R, Dondapati SK, Fenz SF, Schmidt T, Kubick S. Membrane protein synthesis in cell-free systems: From bio-mimetic systems to bio-membranes. FEBS Letters 2014;588: 2774–2781. 10.1016/j.febslet.2014.06.007 24931371

[30] Kuruma Y, Ueda T. The PURE system for the cell-free synthesis of membrane proteins. Nature Protocols 2015;10: 1328–1344. 10.1038/nprot.2015.082 26270393

[31] Matsubayashi H, Kuruma Y, Ueda T. In vitro synthesis of the E. coli sec translocon from DNA. Angewandte Chemie International Edition 2014;53: 7535–7538. 10.1002/anie.201403929 24894900

[32] Mathiasen S, Christensen SM, Fung JJ, Rasmussen SG, Fay JF, Jorgensen SK, et al Nanoscale high-content analysis using compositional heterogeneities of single proteoliposomes. Nature Methods 2014;11: 931–934. 10.1038/nmeth.3062 25086504 PMC4485457

[33] Constantinides PP, Steim JM. Solubility of palmitoyl-coenzyme A in acyltransferase assay buffers containing magnesium ions. Arch. Biochem. Biophys. 1986;250: 267–270. 3767379 10.1016/0003-9861(86)90726-5

[34] Yu X, Liu T, Zhu F, Khosla C. In vitro reconstitution and steady-state analysis of the fatty acid synthase from Escherichia coli. Proceedings of the National Academy of Sciences 2011;108: 18643–18648. 10.1073/pnas.1110852108 22042840 PMC3219124

[35] Tan BK, Bogdanov M, Zhao J, Dowhan W, Raetz CR, Guan Z. Discovery of a cardiolipin synthase utilizing phosphatidylethanolamine and phosphatidylglycerol as substrates. Proceedings of the National Academy of Sciences 2012;109: 16504–16509. 10.1073/pnas.1212797109 22988102 PMC3478633

[36] Arondel V, Benning C, Somerville CR. Isolation and functional expression in Escherichia coli of a gene encoding phosphatidylethanolamine methyltransferase (EC 2.1.1.17) from Rhodobacter sphaeroides. The Journal of Biological Chemistry 1993;268: 16002–16008. 8340421

[37] McFie PJ, Stone SJ. A fluorescent assay to quantitatively measure in vitro acyl CoA:diacylglycerol acyltransferase activity. J. Lipid Res. 2011;52: 1760–1764. 10.1194/jlr.D016626 21653930 PMC3151697

[38] Brea RJ, Cole CM, Devaraj NK. In situ vesicle formation by native chemical ligation. Angewandte Chemie 2014;126: 14326–14329. 10.1002/anie.201408538 25346090 PMC4418804

[39] Hardy MD, Yang J, Selimkhanov J, Cole CM, Tsimring LS, Devaraj NK. Self-reproducing catalyst drives repeated phospholipid synthesis and membrane growth. Proceedings of the National Academy of Sciences 2015;112: 8187–8192. 10.1073/pnas.1506704112 26100914 PMC4500204

[40] Kurihara K, Okura Y, Matsuo M, Toyota T, Suzuki K, Sugawara T. A recursive vesicle-based model protocell with a primitive model cell cycle. Nature Communications 2015;6: 8352 10.1038/ncomms9352 26418735 PMC4598553

[41] Nourian Z, Scott A, Danelon C. Towards the assembly of a minimal divisome. Systems and Synthetic Biology 2014;8: 237–247. 10.1007/s11693-014-9150-x 25136386 PMC4127181

[42] Mercier R, Kawai Y, Errington J. Excess membrane synthesis drives a primitive mode of cell proliferation. Cell 2013;152: 997–1007. 10.1016/j.cell.2013.01.043 23452849

[43] Leirer C, Wunderlich B, Myles V, Schneider M. Phase transition induced fission in lipid vesicles. Biophysical Chemistry 2009;143: 106–109. 10.1016/j.bpc.2009.04.002 19442430

[44] Sakuma Y, Masayuki I. Model system of self-reproducing vesicles. Physical Review Letters 2011;107: 1981101 10.1103/PhysRevLett.107.198101 22181648

[45] Gibson DG, Young L, Chuang RY, Venter JC, Hutchison CA 3rd, Smith HO. Enzymatic assembly of dna molecules up to several hundred kilobases. Nature Methods 2009;6: 343–345. 10.1038/nmeth.1318 19363495

[46] Gibson DG, Smith HO, Hutchison CA 3rd, Venter JC, Merryman C. Chemical synthesis of the mouse mitochondrial genome. Nature Methods 2010;7: 901–903. 10.1038/nmeth.1515 20935651

[47] Tsumoto K, Matsuo H, Tomita M, Yoshimura T. Efficient formation of giant liposomes through the gentle hydration of phosphatidylcholine films doped with sugar. Colloids and Surfaces B: Biointerfaces 2009;68: 98–105. 10.1016/j.colsurfb.2008.09.023 18993037

[48] Astarita G, McKenzie JH, Wang B, Strassburg K, Doneanu A, Johnson J, et al A protective lipidomic biosignature associated with a balanced omega-6/omega-3 ratio in fat-1 transgenic mice. PLoS ONE 2014;9: e96221 10.1371/journal.pone.0096221 24760204 PMC3997567

[49] Gonzalez-Covarrubias V, Beekman M, Uh HW, Dane A, Troost J, Paliukhovich I, et al Lipidomics of familial longevity. Aging Cell 2013;12: 426–434. 10.1111/acel.12064 23451766 PMC3709127

[50] Siegel D, Permentier H, Bischoff R. Controlling detrimental effects of metal cations in the quantification of energy metabolites via ultrahigh pressure-liquid chromatography—electrospray-tandem mass spectrometry by employing acetylacetone as a volatile eluent modifier. Journal of Chromatography A 2013;1294: 87–97. 10.1016/j.chroma.2013.04.029 23643099

