# Supplementary material for: Cell-Free Phospholipid Biosynthesis by Gene-Encoded Enzymes Reconstituted in Liposomes
Source: PLoS One. 2016 Oct 6;11(10):e0163058. doi: 10.1371/journal.pone.0163058 (PMC5053487; doi:10.1371/journal.pone.0163058)
Supplement: S1 File — Supplementary text includes details on (i) lipid handling, (ii) the preparation of lipid standards and calibration curves for absolute quantitation of synthesized lipids, (iii) the calculation of vesicle surface area increase and number of membrane proteins per liposome, and (iv) a description of the main structural and reactivity properties of the phospholipid headgroup modifying enzymes. Table A in S1 File. Optimized parameters for mass spectrometry Multiple Reaction Monitoring (MRM) mode.*The fragmentor voltage was set to 220 mV with PgpA and to 70 mV with PgpC. Table B in S1 File. Overview of the different lipid compositions used. Table C in S1 File. List of primers and sequences used for Gibson assembly or for generating linear DNA templates for IVTT reactions. The lower case three-nucleotide stretch in the DNA sequences denotes the region in the primer where the coding region starts. All sequences are reported in the 5’ to 3’ direction. Figure A in S1 File. Chromatogram profiles of the lipids used in this study. Chromatogram profiles of purified lipids as measured with LC-MS. Lipids DPPA and DOPA were identified using three different fragment ions to facilitate unambiguous detection. Their corresponding m/z of intact and fragment ions are appended in the chromatograms. Figure B in S1 File. Calibration curves for quantifying absolute lipid concentrations. The number of counts for lipid standard samples of known concentrations was plotted against the concentration and a linear fit of the data was performed. For each sample, the concentration error was estimated to be 10% based on the fact that lipids obtained from Avanti Polar Lipids are overpacked by as much as 10% and that standard stocks may experience degradation. The counts error was calculated directly from multiple measurements of lipid standards. Alternatively the error percentage was calculated from multiple injections of synthesized lipids-containing samples as an estimate of the MS data variability on [file pone.0163058.s001.pdf]

SUPPLEMENTARY INFORMATION

Cell-free phospholipid biosynthesis by gene-encoded enzymes reconstituted in liposomes

Andrew Scott, Marek J. Noga, Paul de Graaf, Ilja Westerlaken, Esengul Yildirim and Christophe Danelon*

Department of Bionanoscience, Kavli Institute of Nanoscience, Delft University of Technology, Van der Maasweg 9, 2629 HZ Delft, The Netherlands

Outline

Page 2 **Supplementary Table A**. Optimized parameters for mass spectrometry Multiple Reaction Monitoring (MRM) mode.

Page 3 **Supplementary Table B.** Overview of the different lipid compositions used.

Pages 4-6 **Supplementary Table C.** List of primers and sequences used for Gibson assembly or for generating linear DNA templates for IVTT reactions.

Pages 7-10 **Supplementary Fig. A.** Chromatogram profiles of the lipids used in this study.

Page 11 **Supplementary Fig. B.** Calibration curves for quantifying absolute lipid concentrations.

Page 12 **Supplementary Fig. C.** Experimental workflow for assessing the fraction of synthesized DPPA localized in the liposome membrane.

Page 13 **Supplementary Fig. D.** Addition of EDTA in MS samples leads to higher number of counts for DPPA.

Page 14 **Supplementary Fig. E.** Efficiency of protein separation by the liposome floatation technique as measured by SDS PAGE.

Pages 15-19 **Supplementary text.**

Pages 20-21 **Supplementary references.**

SUPPLEMENTARY TABLES & FIGURES

SUPPLEMENTARY TABLE A

Compound name	Molecular formula	Molecular mass (Da)	Precursor ion m/z [M-H](-)	Product m/z	Fragmentor voltage (V)	Cell accelerator voltage (V)	Polarity	Collision energy (eV)
LPA(16:0)	$C_{19}H_{39}O_7P$	410.24	409.2	152.9	100	7	Negative	13
PA(16:0/16:0)	$C_{35}H_{69}O_8P$	648.47	647.5	152.9	210	7	Negative	33
PA(16:0/16:0)	$C_{35}H_{69}O_8P$	648.47	647.5	255.1	210	7	Negative	29
PA(16:0/16:0)	$C_{35}H_{69}O_8P$	648.47	647.5	391.1	210	7	Negative	17
PE(18:1(9Z)/18:1(9Z))	$C_{41}H_{78}NO_8P$	743.55	742.5	281.2	140	7	Negative	25
PG(18:1(9Z)/18:1(9Z))	$C_{42}H_{79}O_{10}P$	774.54	773.5	281.2	190	7	Negative	37
CL(1'-[18:1(9Z)/18:1(9Z)],3'-[18:1(9Z)/18:1(9Z)])	$C_{81}H_{150}O_{17}P_2$	1457	727.5	281.2	140	7	Negative	29
LPA(18:1)	$C_{21}H_{41}O_7P$	436.26	435.3	152.9	160	7	Negative	17
PA(18:1(9Z)/18:1(9Z))	$C_{39}H_{73}O_8P$	700.5	699.5	281.2	190	7	Negative	37
PA(18:1(9Z)/18:1(9Z))	$C_{39}H_{73}O_8P$	700.5	699.5	153	190	7	Negative	37
PA(18:1(9Z)/18:1(9Z))	$C_{39}H_{73}O_8P$	700.5	699.5	417.2	190	7	Negative	37
PE(16:0/16:0)	$C_{37}H_{74}NO_8P$	691.5	690.5	255.1	230	4	Negative	37
PG(16:0/16:0)	$C_{38}H_{75}O_{10}P$	722.5	721.5	255.2	220 or 70*	4	Negative	45
PS(16:0/16:0)	$C_{38}H_{74}NO_{10}P$	735.51	734.5	255.1	180	4	Negative	41

Table A. Optimized parameters for mass spectrometry Multiple Reaction Monitoring (MRM) mode.

*The fragmentor voltage was set to 220 mV with PgpA and to 70 mV with PgpC.

SUPPLEMENTARY TABLE B

Experiments	Figures	Lipid compositions
Regular	2,3 and 6d	DOPC, DOPE, DOPG, cardiolipin 50.8:35.6:11.5:2.1 in mol. %
Biotinylated liposomes for purification with Dynabeads®	4	Regular supplemented with DSPE-PEG-biotin 0.1% (weight percent)
Proteoliposome purification by floatation	1b	Regular supplemented with DHPE-TRITC 0.5% and DSPE-PEG-biotin 0.5%, both in weight percent
Production DOPA	5c,d	DOPG, DOPE, cardiolipin 54.4:35.6:10 in mol. %
In vesiculo assay	5e,f	DOPC, DOPE, DOPG, cardiolipin 50.8:35.6:11.5:2.1 in mol. % supplemented with TexasRed-DHPE 0.5 % and DSPE-PEG-biotin 1 %, both in weight percent

Table B. Overview of the different lipid compositions used.

SUPPLEMENTARY TABLE C

***plsB* construct**	
**Generating IVTT linear template:**	
plsB fwd:	5’-CATTCGCCATTCAGACTACG-3’
plsB rev:	5’-GACTATGATTACGCCGGTAC-3’
***plsC* construct**	
**Generating IVTT linear template:**	
plsC fwd:	5’-TCGACTCTAGAGGATCTCG-3’
plsC rev:	5’-CCTCAAGACCCGTTTAGAG-3’
***pgpC* construct**	
**Gibson assembly:**	
pgpC fwd: pET11a-EG11371<br>TGGACAGCAAATGGGTCGCGGATCCGGCTGCttgGCAACTCACGAGCGTCG	
comp-pET11a<br>GCAGCCGGATCCGCG	
pgpC rev: pET11a-EG11371<br>AGCAGCCAACTCAGCTTCCTTTCGGGCTTTGctaTTCCAGTTGCTGGAGTTCACC	
comp-pET11a<br>CAAAGCCCGAAAGGAAGCTGA	
**Generating IVTT linear template:**	
HG Fwd:	5’-GGATCTCGACGCTCTCCCTTATG-3’
HG Rev:	5’-GATATCCGGATATAGTTCCTCC-3’
***pgpA* construct**	
**Gibson assembly:**	
pgpA fwd: pET11a-EG10704<br>TGGACAGCAAATGGGTCGCGGATCCGGCTGCatgACCATTTTGCCACGCCA	
comp-pET11a<br>(idem) GCAGCCGGATCCGCG	
pgpA rev: pET11a-EG10704<br>AGCAGCCAACTCAGCTTCCTTTCGGGCTTTGctaCGACAGAATACCCAGCGG	
comp-pET11a<br>(idem) CAAAGCCCGAAAGGAAGCTGA	
**Generating IVTT linear template:**	
HG Fwd:	5’-GGATCTCGACGCTCTCCCTTATG-3’
HG Rev:	5’-GATATCCGGATATAGTTCCTCC-3’
***pgsA* construct**	
**Gibson assembly:**	
pgsA fwd: pET11a-EG10706<br>TGGACAGCAAATGGGTCGCGGATCCGGCTGCatgCAATTTAATATCCCTACGTTGCTTACAC	
comp-pET11a<br>(idem) GCAGCCGGATCCGCG	
pgsA rev: pET11a-EG10706<br>AGCAGCCAACTCAGCTTCCTTTCGGGCTTTGtcaCTGATCAAGCAAATCTGCACGC	
comp-pET11a<br>(idem) CAAAGCCCGAAAGGAAGCTGA	

Generating IVTT linear template:

HG Fwd: 5'-GGATCTCGACGCTCTCCCTTATG-3'

HG Rev: 5'-GATATCCGGATATAGTTCCTCC-3'

***psd* construct**

Gibson assembly:

psd fwd: pET11a-EG10775
TGGACAGCAAATGGGTCGCGGATCCGGCTGCttgTTAAATTCATTTAAACTTTCGCTACAGTACATTCTGC

comp-pET11a
(idem) GCAGCCGGATCCGCG

psd rev: pET11a-EG10775
AGCAGCCAACTCAGCTTCCTTTCGGGCTTTGttaGACCTGGTCTTTTTTGTCGTCAACCA

comp-pET11a
(idem) CAAAGCCCGAAAGGAAGCTGA

Generating IVTT linear template:

HG Fwd: 5'-GGATCTCGACGCTCTCCCTTATG-3'

HG Rev: 5'-GATATCCGGATATAGTTCCTCC-3'

***pssA* construct**

Gibson assembly:

pssA fwd: pET11a-EG110781
TGGACAGCAAATGGGTCGCGGATCCGGCTGCatgTTGTCAAAATTTAAGCGTAATAAACATCAACAAC

comp-pET11a
(idem) GCAGCCGGATCCGCG

pssA rev: pET11a-EG110781
AGCAGCCAACTCAGCTTCCTTTCGGGCTTTGttaCAGGATGCGGCTAATTAATCGGT

comp-pET11a
(idem) CAAAGCCCGAAAGGAAGCTGA

Generating IVTT linear template:

HG Fwd: 5'-GGATCTCGACGCTCTCCCTTATG-3'

HG Rev: 5'-GATATCCGGATATAGTTCCTCC-3'

***cdsA* construct**

Gibson assembly:

cdsA fwd: pET11a-EG10139
TGGACAGCAAATGGGTCGCGGATCCGGCTGCttgCTGAAGTATCGCCTGATATCTGC

comp-pET11a
(idem) GCAGCCGGATCCGCG

cdsA rev: pET11a-EG10139
AGCAGCCAACTCAGCTTCCTTTCGGGCTTTGttaAAGCGTCCTGAATACCAGTAACAACAAG

comp-pET11a
(idem) CAAAGCCCGAAAGGAAGCTGA

Generating IVTT linear template:

HG Fwd: 5'-GGATCTCGACGCTCTCCCTTATG-3'

HG Rev: 5'-GATATCCGGATATAGTTCCTCC-3'

Table C. List of primers and sequences used for Gibson assembly or for generating linear DNA templates for IVTT reactions. The lower case three-nucleotide stretch in the DNA sequences denotes the region in the primer where the coding region starts. All sequences are reported in the 5' to 3' direction.

SUPPLEMENTARY FIGURE A

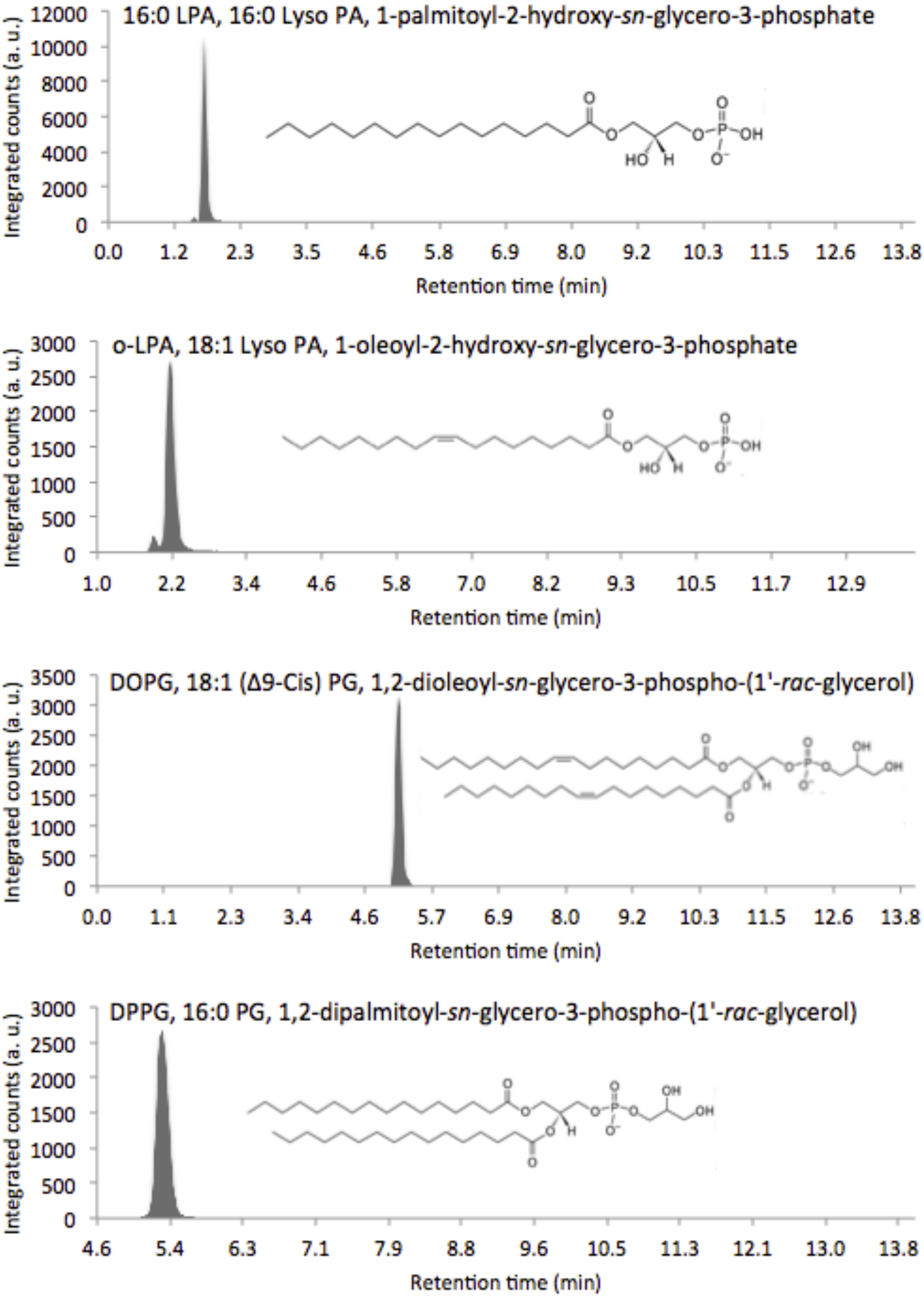

SUPPLEMENTARY FIGURE A (CONTINUED)

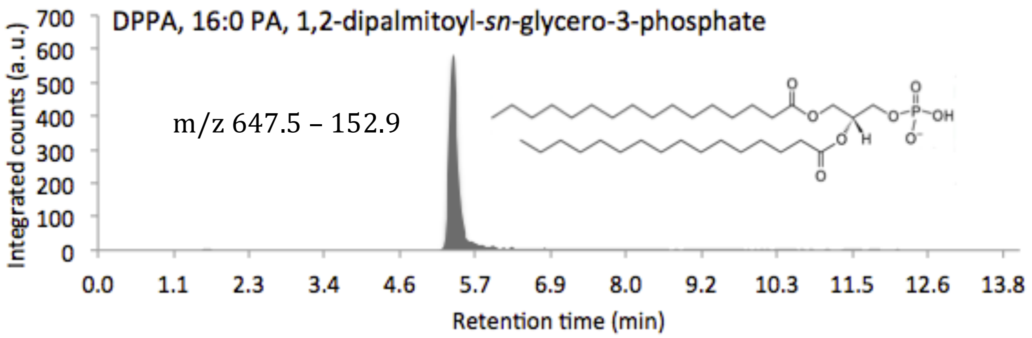

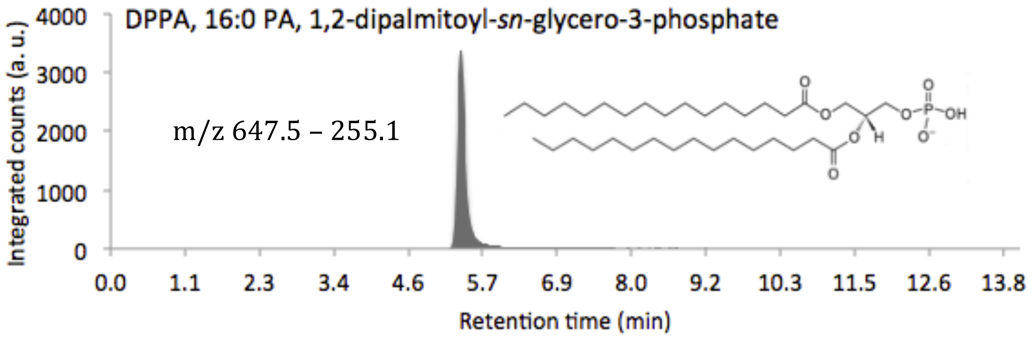

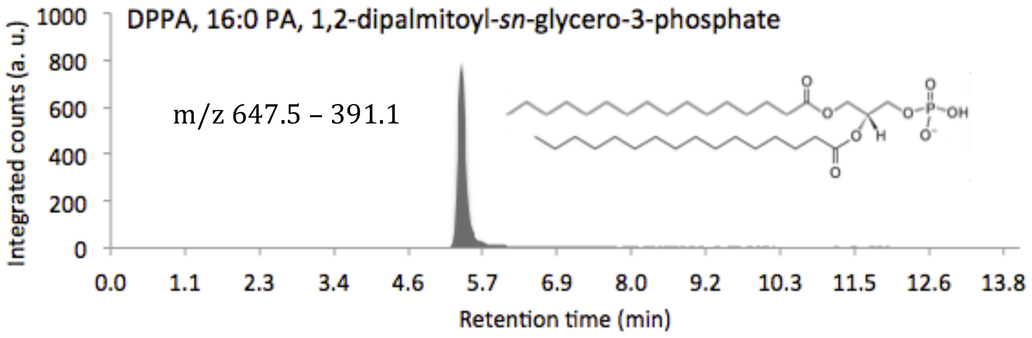

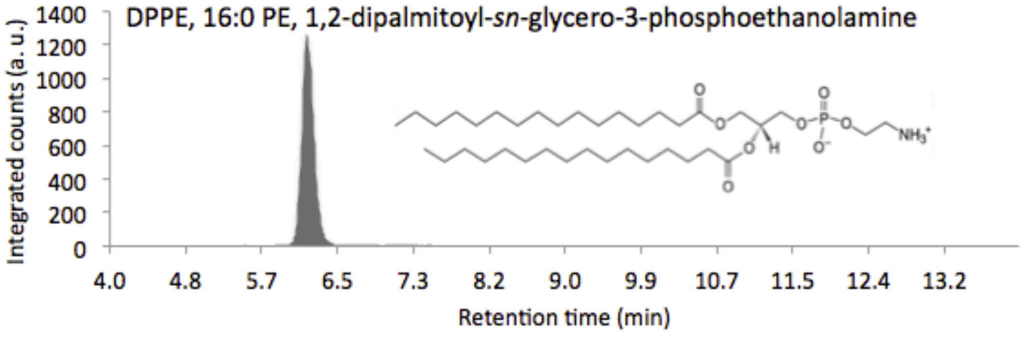

SUPPLEMENTARY FIGURE A (CONTINUED)

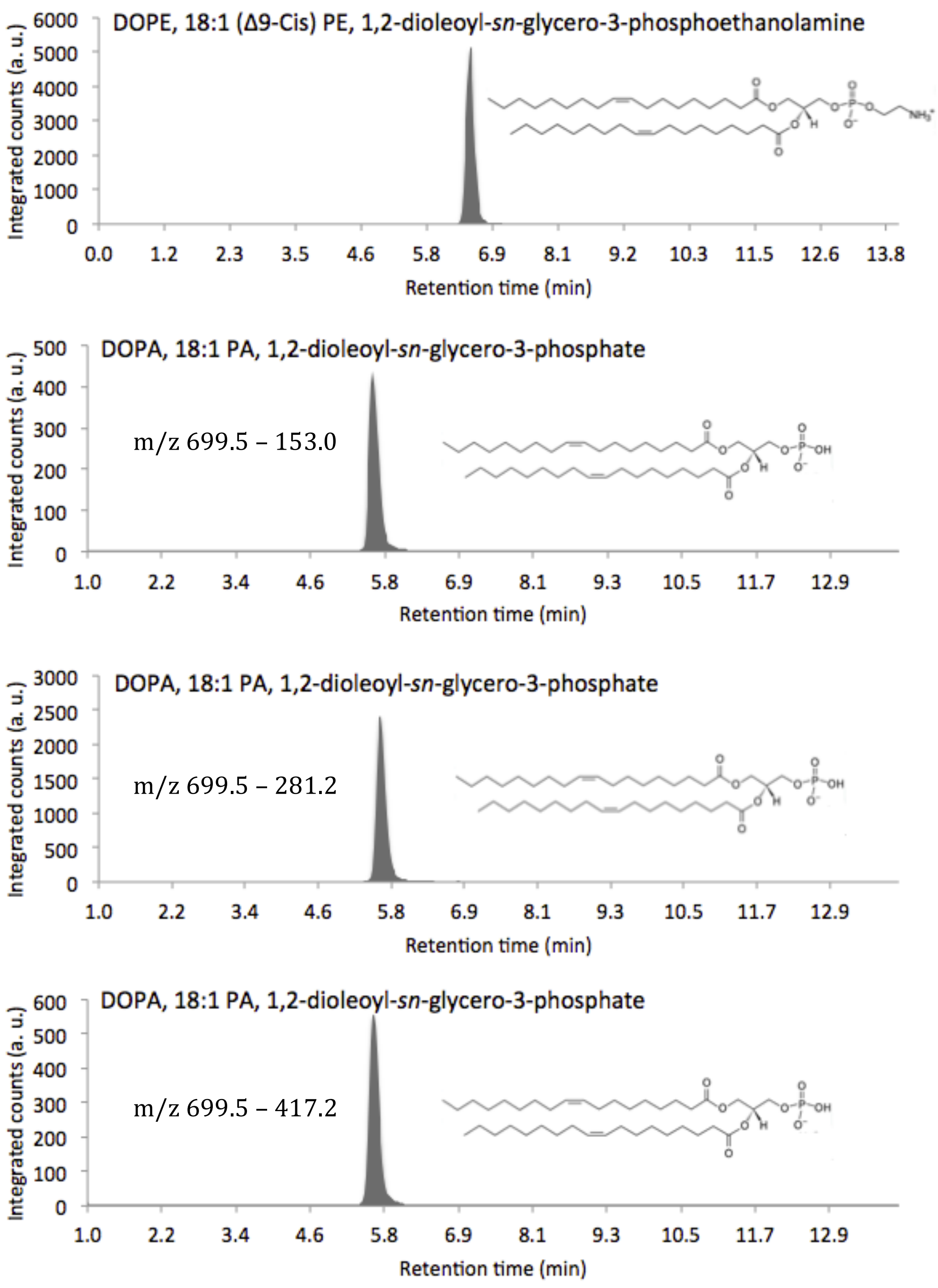

SUPPLEMENTARY FIGURE A (CONTINUED)

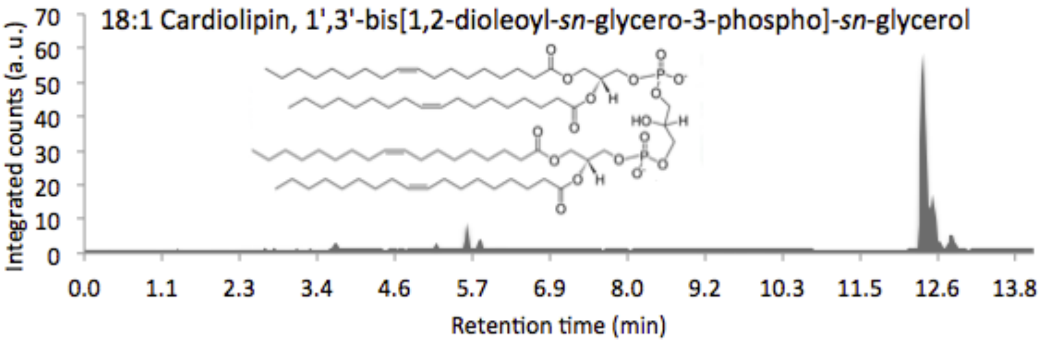

Figure A. Chromatogram profiles of the lipids used in this study. Chromatogram profiles of purified lipids as measured with LC-MS. Lipids DPPA and DOPA were identified using three different fragment ions to facilitate unambiguous detection. Their corresponding m/z of intact and fragment ions are appended in the chromatograms.

SUPPLEMENTARY FIGURE B

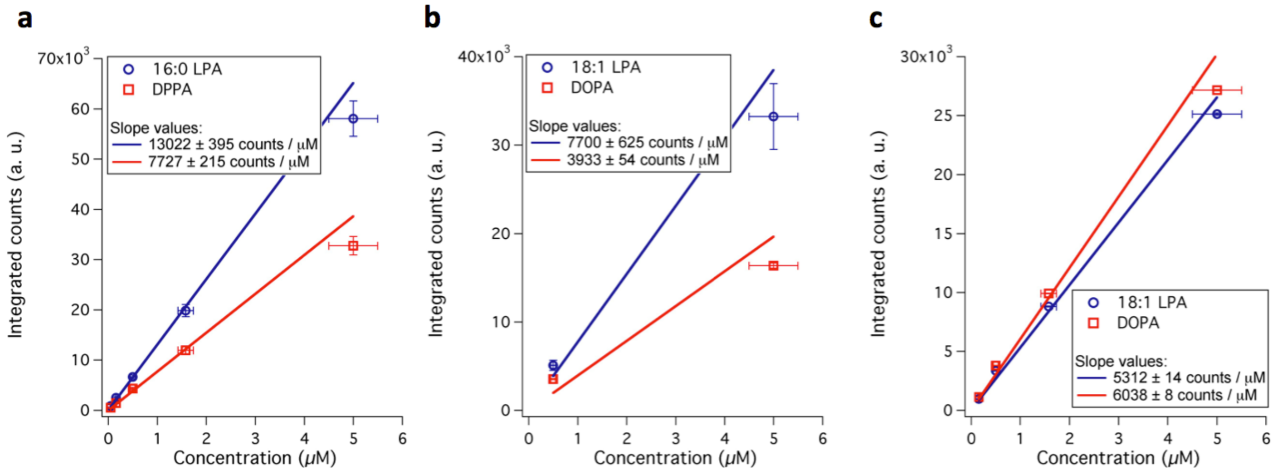

Figure B. Calibration curves for quantifying absolute lipid concentrations. The number of counts for lipid standard samples of known concentrations was plotted against the concentration and a linear fit of the data was performed. For each sample, the concentration error was estimated to be 10% based on the fact that lipids obtained from Avanti Polar Lipids are overpacked by as much as 10% and that standard stocks may experience degradation. The counts error was calculated directly from multiple measurements of lipid standards. Alternatively the error percentage was calculated from multiple injections of synthesized lipids-containing samples as an estimate of the MS data variability on a given day. The concentration to counts conversion factors are calculated from the slopes divided by ten to account for the ten-fold dilution of samples before injection to the MS. (**a**) Calibration plots for the kinetics measurements presented in Figure 3b-e. Values of conversion factors are 1302 cts/µM for LPA and 778 cts/µM for DPPA. (**b**) Calibration plots for the oleoyl-CoA bulk experiments presented in Figure 5c,d. Values of conversion factors are 770 cts/µM for 18:1 LPA and 393 cts/µM for DOPA. (**c**) Calibration plots for the oleoyl-CoA *in vesiculo* experiments presented in Figure 5e,f. Values of conversion factors are 531 cts/µM for 18:1 LPA and 604 cts/µM for DOPA. Calibration curves were performed on the same day as measurements on stored samples to minimize differences observed when experiments were made on different days. This, together with contribution from the fact that preparation of lipid standards was different for the three sets of experiments (Supplementary text), explains the different conversion factor values obtained in b) and c).

SUPPLEMENTARY FIGURE C

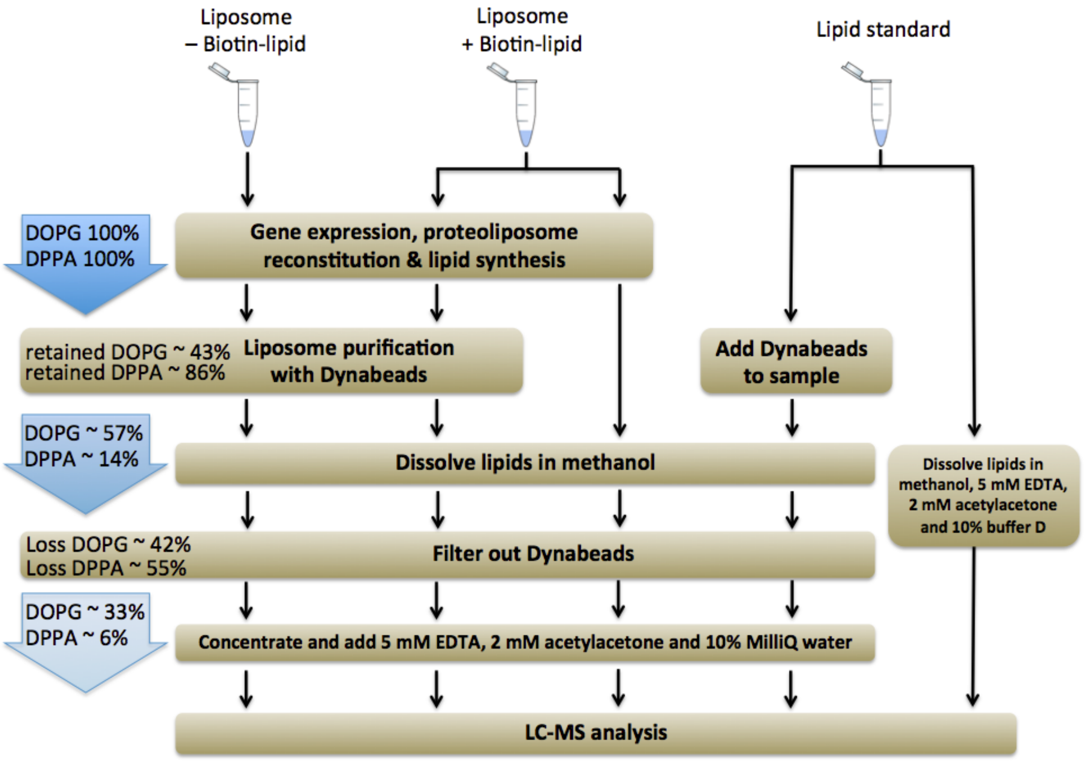

Figure C. Experimental workflow for assessing the fraction of synthesized DPPA localized in the liposome membrane. The loss of lipids during the filtration step was determined by measuring the number of counts of lipid standards (DPPA and DOPG, three concentrations each) treated with or without filtration. Linearity over the used concentration range was validated for both lipids and both treatments (not shown). For each lipid the loss introduced by filtering was calculated as: Loss % = slope $_{\text{filtered}}$ / slope $_{\text{unfiltered}}$ x 100. Values of 42% and 55% were obtained for DOPG and DPPA, respectively. The fraction of DOPG and DPPA lipid retained during purification was assessed using biotin-labelled vesicles and subjecting, or not, samples to purification with Dynabeads. Recovered lipid values correspond to 57% and 14% for DOPG and DPPA, respectively. The sample devoid of biotinylated lipid served to infer the loss of DPPA due to nonspecific adsorption to the magnetic beads or to the tube during purification. The corresponding count number was subtracted from that of the biotin-labelled vesicle sample to determine the actual fraction of DPPA that was retained through liposome immobilization. In Figure 4, we estimated this fraction to represent ~15% of the total DPPA synthesized, which after correcting for the fact that only ~52% of internal standard DOPG is recovered leads to ~30%.

SUPPLEMENTARY FIGURE D

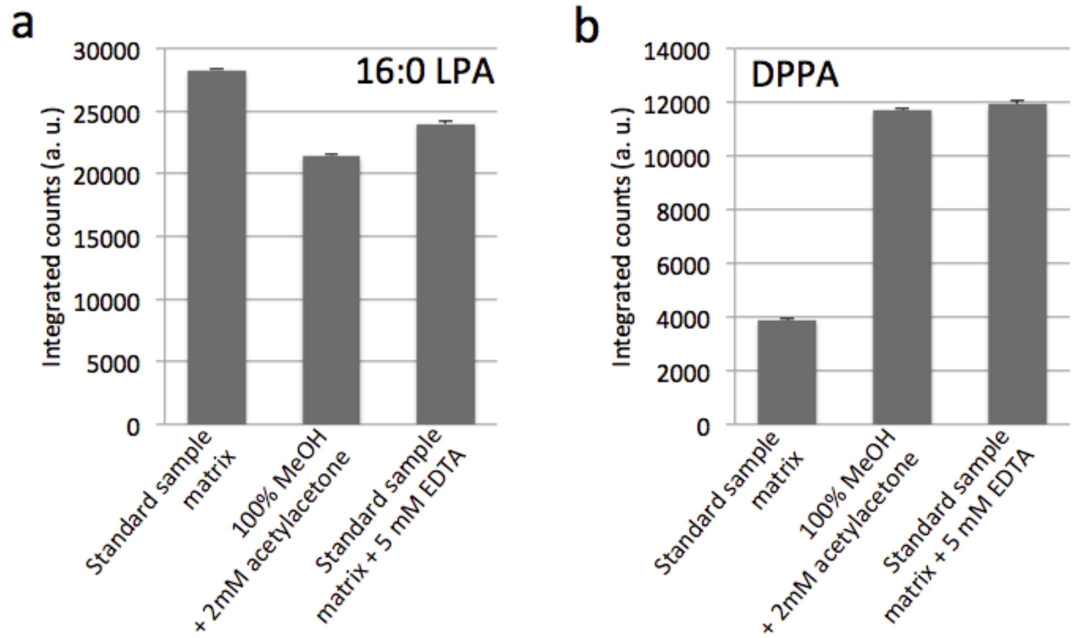

Figure D. Addition of EDTA in MS samples leads to higher number of counts for DPPA. A higher number of counts for DPPA was observed when standard solutions were supplemented with 5 mM EDTA. These results suggest that interaction between the charged phosphate group of the lipids, metal ions and exposed silica in the column could lower the absolute number of counts. We therefore included EDTA in some samples (*in vesiculo*, headgroup modification and Dynabeads purification experiments) to increase signal and reproducibility. Standard sample matrix corresponds to 90% methanol with 2 mM acetylacetone and 10% buffer D.

SUPPLEMENTARY FIGURE E

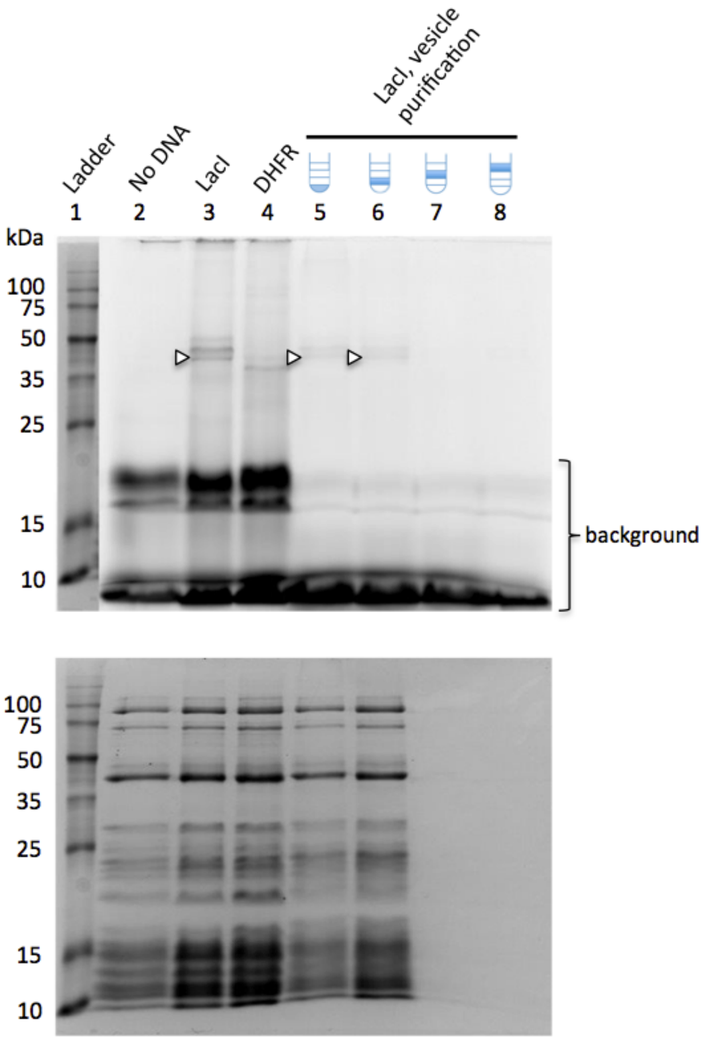

Figure E. Efficiency of protein separation by the liposome floatation technique as measured by SDS PAGE. PURE*frex* reactions were carried out as described in the Methods section. Production of the LacI and DHFR proteins was used as a control for removal of non-membrane-bound proteins. The same SDS PAGE was analysed through fluorescence of nonnatural amino acids as a marker of translation products (top image) and by CBB staining as a total protein marker (bottom image). Lane 1, V849a protein ladder; Lane 2, no DNA non-purified; Lane 3, LacI non-purified; Lane 4, DHFR non-purified; Lane 5, LacI purified, bottom ¼; Lane 6, Lac I purified, second ¼; Lane 7, LacI purified, third ¼; Lane 8, LacI purified, top ¼. Arrowheads indicate the synthesized LacI protein in the unpurified and bottom ½ fractions of the purified samples. Good removal of bulk protein is achieved by harvesting the top ½ of the sample, as can be seen from the protein-free lanes 7 and 8. Hence, isolation of membrane-bound GPAT and LPAAT proteins that co-purified with liposomes was ensured by harvesting a sample fraction < ¼ from the top (Figure 1b).

SUPPLEMENTARY TEXT

Lipid handling

Lipids dissolved in chloroform (Avanti Polar Lipids) were aliquoted in PTFE topped glass vials using Hamilton syringes and stored at –20 °C. Subsequently lipids were handled with Gilson Microman positive displacement pipettes using capillary piston tips. Compounds LPA, DPPA and acyl-CoAs were purchased as dried powders. Whole amounts as purchased were dissolved in appropriate solvent (chloroform:methanol:water at vol. ratio 65:35:8 for LPA and DPPA, or 80:20:2 for acyl-CoAs,), then aliquoted in PTFE topped vials with Hamilton syringes, dried overnight at room temperature and pressure, and stored at –20 °C. Individual aliquots were re-dissolved in appropriate solvent at 0.1 or 1 mg/mL stock concentrations. Lipids received in organic solvent were directly aliquoted and stored at –20 °C.

Preparation of lipid standards and calibration curves for absolute quantitation of synthesized lipid concentrations

Calibration measurements were performed to determine the absolute concentration of 16:0 LPA, 18:1 LPA, DPPA and DOPA produced in some experiments (Figures 3b-e and 5). Standard samples were prepared by serial dilution of stock concentrations of lipids in their respective organic solvent mixture used for storage. For the kinetics experiments, 2 µL of 16:0 LPA and DPPA standard solutions was added to 3 µL of PURE*frex* system (containing 10 ng/µL of *plsB* and *plsC* DNA) with 0.4 mg/mL SUVs of regular composition, 5 mM β-mercaptoethanol, 500 µM G3P and 0.4 U/µL RNase inhibitor in order to reproduce the same lipid background than that in the measured IVTT reaction samples. Samples were further diluted with 25 µL methanol containing 2 mM acetylacetone to a final concentration of 5 µM. The standard solutions were then centrifuged at 16,100 rcf (Eppendorf 5415R) and 5 µL of the supernatants were injected in the LC-MS system. For experiments with oleoyl lipids in the outside-out proteoliposome configuration, 18:1 LPA and DOPA stock solutions in chloroform were diluted in methanol with 2 mM acetylacetone. For *in vesiculo* experiments, standards were prepared by mixing SUVs, 18:1 LPA and DOPA in 90% methanol with 2 mM acetylacetone and 5 mM EDTA (see section on EDTA), 10% buffer D (20 mM HEPES, 14 mM magnesium acetate, 180 mM potassium glutamate, pH 7.6) to final concentrations of 2.1 mg/mL liposome and 5 µM 18:1 LPA and DOPA. The standards were finally diluted with a solution of methanol with 2 mM acetylacetone and 5 mM EDTA.

Calibration curves for the different lipid standards were obtained by plotting the number of integrated counts against concentration and a linear fit of the data was performed using IGOR Pro

(WaveMetrics) (Supplementary Figure B). The concentration of synthesized lipids in reaction samples was determined by reporting the calculated peak integrated counts on the calibration curve and correcting for the 10-time dilution of the samples (see Methods). In order to correct for inherent pipetting errors, the amount of DOPG included in the initial vesicle composition was used as an internal standard. The number of counts measured for each synthesized lipid (*C*t) was normalized by the number of counts for DOPG (C_{DOPG}):

$$Ct_{norm} = \frac{Ct}{C_{DOPG}}.$$

The mean and standard deviation of the normalized counts for each data point were calculated. Concentrations were obtained by multiplying the normalized counts by the average concentration of DOPG and solving the line equation for concentration:

$$Conc = \frac{Ct_{norm} \cdot \overline{DOPG}}{b},$$

where *b* is the slope. Concentration errors were calculated by using the variance formula:

$$\sigma_{conc} = \sqrt{\left(\frac{\overline{DOPG}}{b}\right)^2 \sigma_{Ctnorm}^2 + \left(\frac{\overline{Ctnorm}}{b}\right)^2 \sigma_{\overline{DOPG}}^2 + \left(\frac{\overline{Ctnorm} \cdot \overline{DOPG}}{b^2}\right)^2 \sigma_b^2}.$$

An alternative method was used to convert MS counts into lipid concentrations. First, a linear regression of the count ratios DPPA/DOPG to the known concentrations of DPPA was made:

$$\frac{DPPA_c}{DOPG_c} = b \cdot [DPPA].$$

The slope *b* and its error σ_b were extracted. We then calculated the actual value of the concentrations in a sample as:

$$[DPPA] = \frac{DPPA_c}{DOPG_c} / b.$$

The error of DPPA counts was calculated as:

$$\sigma_{DPPA} = \sqrt{\left(\frac{1}{b}\right)^2 \sigma_{\frac{DPPA_c}{DOPG_c}}^2 + \left(\frac{\frac{DPPA_c}{DOPG_c}}{b^2}\right)^2 \sigma_b^2}.$$

Similar results were obtained as with the first method.

Calculation of increase surface area of liposomes upon incorporation of synthesized DPPA

To estimate the vesicle growth through synthesis and membrane incorporation of DPPA lipids, the following calculation was made. First the initial surface area, *A*, of a 400-nm-sized vesicle was calculated:

$$A = 4 \times \pi \times (200 \times 1e^{-9})^2.$$

Then the total number of lipids per vesicles, $N_{lip./ves.}$, was calculated taking into account the two leaflets of the membrane. A cross-sectional area of 72.1 Å^2 corresponding to that of a DOPC molecule was assumed.

$$N_{lip./ves.} = 2 \times A / (72.1 \times (1e^{-10})^2).$$

The concentration of vesicles is derived from the initial concentration of lipids, $C_{lip.} = 508$ µM, and the number of lipids per liposome as:

$$C_{ves.} = C_{lip.} / N_{lip./ves.}$$

The concentration of synthesized DPPA was determined by kinetics experiments with both GPAT and LPAAT enzymes and a value of 26 µM was found (Figure 3e). Given that about 28% of total synthesized DPPA integrated in liposome membrane (Figure 4d), the concentration of membrane-inserted DPPA, C_{PA}, is 7 µM. Therefore, assuming homogenous partitioning of DPPA lipids between vesicles, the number of DPPA molecules per liposome is:

$$N_{PA/ves.} = C_{PA} / C_{ves.}.$$

Using a cross-sectional area per DPPA lipid of 50 Å^2 it is possible to calculate the total additional surface area as:

$$A_{PA/ves.} = 50 \times (1e^{-10})^2 \times N_{PA/ves.} / 2.$$

The percentage area increase was calculated as $A_{PA/ves.} / A \times 100$ and a value of ~1% was found.

Estimation of the number of synthesized membrane proteins per liposome

Based on previous PURE*frex*-based IVTT experiments we estimate the concentration of synthesized proteins to be in the order of 0.5 µM[1,2]. The precise concentration may vary from one protein to the other, the fraction of active proteins too. An approximation of the number of synthesized proteins and the number of reconstituted pathways per vesicle can be found assuming that (i) an amount of 500 nM protein is produced from a single-gene expression reaction, hence co-expression of 5 genes will generate ~100 nM of each specific protein (though a nonlinear relationship is expected but difficult to predict), (ii) 1/3 of proteins incorporates inside liposomes (see Figure 1c where the fraction of GPAT and LPAAT proteins co-purified with vesicles can be evaluated), (iii) proteins randomly insert into liposomes, such that the surface density of proteins is the same for every liposome, (iv) all membrane-bound proteins are active and (v) proteins can insert into the bilayer in a bi-directional manner (this might not be the case for PssA) limiting the fraction of active enzymes to 0.5. Under these conditions one liposome contains ~225 membrane proteins. This means that every liposome potentially contains ~45 copies of the 5-protein pathways. Further quantitative investigations are definitely needed to refine these numbers, a major challenge being to determine the fraction of active proteins.

Phospholipid headgroup modification enzymes

Besides complex functions such as facilitating compartment division, regulating transport of molecules and participating to membrane signaling, the most basic requirement of the lipid membrane is the ability to form stable bilayers. Given the inverse cone-shape structure (ratio of the diameter of the headgroup to that of the tails is < 1) of PA glycerophospholipids, such as DPPA and DOPA used in this study, they do not pack in a flat lipid sheet and, thus, cannot form stable bilayers in regular conditions. As a comparison diacyl-PC lipids, which are most frequently used to form supported bilayers or liposomes, have a cylinder shape with headgroup-to-tails diameter ratio ~1. Therefore, converting the PA lipids synthesized in the vesicles into phospholipids of larger head group is essential to support stability of growing liposome-based minimal cells.

It is widely reported that the composition of *E. coli* membranes is approximately 80% diacylphosphatidylethanolamine (XXPE) and 20% diacylphosphatidylglycerol (XXPG) with a small fraction of cardiolipin[3,4,5]. Hence, it is natural to convert PA lipids into PE and PG in the *E. coli*-based PURE system to mimic a physiological membrane environment. The enzymes required for the synthesis of PE and PG lipids from PA are shown in Figure 6. Some of their structural and functional properties are summarized below.

CdsA. The first reaction of the pathway for both the synthesis of PE and PG lipids is where a phosphatidic acid, cytidine triphosphate (CTP) and a proton react to form a cytosine diphosphate-diacylglycerol with the release of diphosphate. This reaction is catalyzed by the gene product of *cdsA*, a phosphatidate cytidylyltransferase. The molecular weight of the protein as predicted by the nucleotide sequence is ~31 kDa and the experimentally observed molecular weight is ~27 kDa[6]. Sequence analysis predicts eight transmembrane helices and databases list it as localized to the inner membrane of *E. coli*[7].

PssA. The first committed step of synthesizing PE from PA is the formation of phosphatidylserine. The gene product of *pssA*, the diacylphosphatidylserine synthase, ligates L-serine to CDP-diacylglycerol via a reaction mechanism releasing CMP and a proton[8,9]. The enzyme PssA is considered to be a peripheral membrane protein that spends time associated with membranes or free in the cytoplasm[10]. The predicted molecular weight from the nucleotide sequence is ~53 kDa[11].

Psd. The final step in the formation of PE lipids is the decarboxylation of phosphatidylserine by the gene product of *psd*, the phosphatidylserine decarboxylase. In this reaction

diacylphosphatidylserine (PS) and a proton react, and diacylphosphatidylethanolamine and carbon dioxide are released. The enzyme itself is a heterodimer, produced from a single polypeptide that self-cleaves posttranslationally[12,13]. The Psd enzyme is located at the inner membrane[14,15]. The predicted molecular weight from the nucleotide sequence is ~36 kDa[16].

PgsA. The first committed step in the PG biosynthesis pathway starting from phosphatidic acid is the formation of phosphatidylglycerolphosphate (PGP). The gene product of *pgsA*, the phosphatidylglycerophosphate synthase, accepts CDP diacylglycerol and ligates the *sn*-glycerol-3-phosphate to it releasing diacyl-phosphatidylglycerolphosphate, CMP and a proton. PgsA is an integral membrane protein[9] located at the inner membrane[13]. The enzyme has an absolute requirement for magnesium to function[17] (PURE*frex* contains 14 mM Mg^{2+}). The predicted molecular weight from the nucleotide sequence is ~21 kDa[18].

PgpA, PgpC (PgpB). The final step in the formation of PG lipids is the dephosphorylation of PGP into diacyl-phosphatidylglycerol. There exist three enzymes, called phosphatidylglycerolphosphatases, that can perform this hydrolysis reaction[19]. They are the products of the *pgpA*, *pgpB* and *pgpC* genes. PgpA contains a single transmembrane segment and its active site faces the cytoplasm[20]. PgpB was originally thought to be an outer membrane phosphatase, but more recent results indicate an inner membrane location[21]. PgpC is predicted to have a single transmembrane domain with its active site facing the cytoplasm[19]. The molecular weight of PgpA from its nucleotide sequence is ~19 kDa[22], that of PgpB is ~29 kDa[23], (while from experiment it is ~28 kDa[22]) and that of PgpC is ~24 kDa[24]. In our experiments we expressed either PgpA or PgpC, both resulting in synthesis of PG lipids.

To our knowledge we are the first to attempt to express and study the complete synthesis pathways for PE and PG lipids *in vitro*. Previous works focused on generating phosphatidylcholine with purified proteins[25,26]. Here we show that we can produce the membrane forming lipids PG and PE as well as their intermediates using cell-free synthesized enzymes and simple G3P and mono-acyl-CoA precursors.

REFERENCES

[1] Shimizu, Y., Kuruma, Y., Kanamori, T. & Ueda, T. The PURE system for protein production. *Methods in Molecular Biology* **1118**, 275–284 (2014).

[2] van Nies, P., Soler Canton, A., Nourian, Z. & Danelon, C. Monitoring mRNA and protein levels in bulk and in model vesicle-based artificial cells. *Methods in Enzymology* **550**, 187–214 (2015).

[3] Dowhan, W. Molecular basis for membrane phospholipid diversity: why are there so many lipids? *Annual Review of Biochemistry* **66**, 199–323 (1997).

[4] Oursel, D. et al. Lipid composition of membranes of *escherichia coli* by liquid chromatography/tandem mass spectrometry using negative electrospray ionization. *Rapid communications in mass spectrometry* **21**, 1721–1728 (2007).

[5] Furse, S., Wienk, H., Boelens, R., de Kroon, A. I. & Killian, J. A. *E. coli* mg1655 modulates its phospholipid composition through the cell cycle. *FEBS letters* **589**, 2726–2730 (2015).

[6] Sparrow, C. P. & Raetz, C. R. Purification and properties of the membrane-bound CDP diglyceride synthetase from Escherichia coli. *Journal of Biological Chemistry* **260**, 12084–12091 (1985).

[7] UniProt. Uniprotkb - p0abg1 (cdsa ecoli) (2015). URL, http://www.uniprot.org/uniprot/P0ABG1.

[8] Raetz, C. R. & Kennedy, E. P. The association of phosphatidylserine synthetase with ribosomes in extracts of *escherichia coli*. *Journal of Biological Chemistry* **247**, 2008–2014 (1972).

[9] Dowhan, W. A retrospective: use of *escherichia coli* as a vehicle to study phospholipid synthesis and function. Biochimica et Biophysica Acta (BBA)-*Molecular and Cell Biology of Lipids* **1831**, 471–494 (2013).

[10] Louie, K. & Dowhan, W. Investigations on the association of phosphatidylserine synthase with the ribosomal component from *escherichia coli*. *Journal of Biological Chemistry* **255**, 1124–1127 (1980).

[11] EcoCyc. Pssa - eg10781 (2015). URL http://www.ecocyc.org

[12] Li, Q.-X. & Dowhan, W. Structural characterization of *escherichia coli* phosphatidylserine decarboxylase. *Journal of Biological Chemistry* **263**, 11516–11522 (1988).

[13] Dowhan, W. Phosphatidylglycerophosphate synthase from *escherichia coli*. *Methods in enzymology* **209**, 313–321 (1992).

[14] Bell, R. M., Mavis, R. D., Osborn, M. & Vagelos, P. R. Enzymes of phospholipid metabolism: localization in the cytoplasmic and outer membrane of the cell envelope of *escherichia coli* and *salmonella typhimurium*. *Biochimica et Biophysica Acta (BBA)-Biomembranes* **249**, 628–635 (1971).

[15] White, D. A., Albright, F. R., Lennarz, W. & Schnaitman, C. A. Distribution of phospholipid-synthesizing enzymes in the wall and membrane subfractions of the envelope of *escherichia coli*. *Biochimica et Biophysica Acta (BBA)-Biomembranes* **249**, 636–642 (1971).

[16] EcoCyc. Psda -eg10775 (2015). URL http://www.ecocyc.org

[17] Hirabayashi, T., Larson, T. J. & Dowhan, W. Membrane-associated phosphatidylglycerophosphate synthetase from *escherichia coli*: purification by substrate affinity chromatography on cytidine 5'-diphospho-1, 2-diacyl-sn-glycerol sepharose. *Biochemistry* **15**, 5205–5211 (1976).

[18] EcoCyc. Psda -eg10706 (2015). URL http://www.ecocyc.org

[19] Icho, T. & Raetz, C. Multiple genes for membrane-bound phosphatases in *escherichia coli* and their action on phospholipid precursors. *Journal of bacteriology* **153**, 722–730 (1983).

[20] Lu, Y.-H., Guan, Z., Zhao, J. & Raetz, C. R. Three phosphatidylglycerol-phosphate phosphatases in the inner membrane of *escherichia coli*. *Journal of Biological Chemistry* **286**, 5506–5518 (2011).

[21] Touze, T., Blanot, D. & Mengin-Lecreulx, D. Substrate specificity and membrane topology of *escherichia coli* pgpb, an undecaprenyl pyrophosphate phosphatase. *Journal of Biological Chemistry* **283**, 16573–16583 (2008).

[22] Icho, T. Membrane-bound phosphatases in *escherichia coli*: sequence of the pgpa gene. *Journal of bacteriology* **170**, 5110–5116 (1988).

[23] EcoCyc. Ppgb - eg10705 (2015). URL http://www.ecocyc.org

[24] EcoCyc. Ppgc - eg11371 (2015). URL http://www.ecocyc.org

[25] Deamer, D. W. & Gavino, V. Lysophosphatidylcholine acyltransferase: purification and applications in membrane studies. *Annals of the New York Academy of Sciences* **414**, 90–96 (1983).

[26] Schmidli, P. K., Schurtenberger, P. & Luisi, P. L. Liposome-mediated enzymatic synthesis of phosphatidylcholine as an approach to self-replicating liposomes. *Journal of the American Chemical Society* **113**, 8127–8130 (1991).
